# Risk stratification for long-term inpatient costs in mental disorders: a dual-track machine learning approach using baseline EHRs and hospitalization trajectories

**DOI:** 10.1186/s12913-026-14274-y

**Published:** 2026-02-28

**Authors:** Mengge Zhang, Guoliang Pan, Haohui Shen, Xiuwen He, Jingyi Xiang, Simeng Wang, Mingyang Yao, Yilong Yang

**Affiliations:** 1 Department of Health Policy and Management, School of Public Administration, Hangzhou Normal University, No.2318 Yuhangtang Road, Yuhang District, Hangzhou, Zhejiang 311121 China; 2 Shenyang Mental Health Center, Shenyang, Liaoning China; 3 Institute of Health Professions Education Assessment and Reform, China Medical University, Shenyang, Liaoning China

**Keywords:** Mental disorders, Hospitalization costs, Machine learning, Trajectory clustering, Electronic health records

## Abstract

**Background:**

Mental disorders (MDs) impose substantial long-term inpatient costs, yet existing prediction models rarely account for dynamic hospitalization trajectories or diagnostic heterogeneity. This study developed and validated a dual-track machine learning framework integrating baseline features with trajectory-derived patterns to predict three-year cumulative hospitalization costs for patients with MDs in China.

**Methods:**

We conducted a retrospective cohort study using electronic health records from 3,396 adults with first admission to a psychiatric hospital (2017–2018) and three‑year follow‑up. State sequence analysis and hierarchical clustering identified distinct hospitalization trajectory patterns. Ten baseline variables available at index admission (Set A) and trajectory cluster membership (Set B) were used to train five regression models with stratified 70:30 split and five‑fold cross‑validation. Performance was evaluated using R², RMSE, and MAE on log‑transformed costs. SHAP (SHapley Additive exPlanations) analysis was applied to interpret the optimal model and examine diagnostic heterogeneity.

**Results:**

Four distinct trajectory patterns were identified: low‑frequency short‑stay (64.7%), high‑frequency short‑stay (10.0%), long‑term intermittent (4.8%), and long‑term continuous (20.5%). The gradient boosting machine (GBM) achieved the best test performance using Set A (R² = 0.35), significantly outperforming linear regression (R² = 0.33) and random forest (R² = 0.31). Adding trajectory clusters (Set B) increased R² to 0.71 (ΔR² = 0.36), indicating strong association between long‑term hospitalization patterns and cumulative costs, though this component is only retrospectively explanatory. SHAP identified Payment methods, aCCI, Diagnosis groups, and Age as dominant cost drivers. Model performance was stable for the F2 group (61.8% of cohort) but markedly lower for rare diagnostic subgroups (F0, F1).

**Conclusions:**

Risk stratification for three‑year cumulative hospitalization costs is feasible using only routine baseline information from first admission. The proposed dual‑track framework separates prospective prediction from retrospective explanation, providing a methodologically sound tool for institutional resource planning and high‑risk screening in mental health settings. Future work requires external validation and implementation studies.

**Supplementary Information:**

The online version contains supplementary material available at 10.1186/s12913-026-14274-y.

## Introduction

Mental disorders (MDs) are a serious global public health challenge, affecting approximately 1 billion people, or 13% of the world’s population [1]. These conditions are characterized by high prevalence, a prolonged course, and a tendency to relapse. Consequently, they have become a leading cause of disease burden and disability, accounting for 6.8% of global disability-adjusted life years (DALYs) [2]. In China, the prevalence of MDs (16.6%) is higher than the global average [3]. The years lived with disability (YLDs) attributable to these disorders rank second among all diseases nationwide. According to World Health Organization estimates, MDs are projected to cause economic losses exceeding $9 trillion in China between 2012 and 2030, including both direct medical costs and indirect productivity losses [4]. Frequent relapse often leads to repeated hospitalizations. Therefore, inpatient costs constitute a major part of mental health service expenditures, placing a shared financial burden on medical insurance funds, healthcare institutions, and patients’ families [5]. Currently, nearly 90% of China’s mental health services are delivered by specialized psychiatric institutions, forming a service model dominated by specialized hospitals [6]. Within this system, regional tertiary psychiatric hospitals primarily treat patients with severe, complex, or long-term conditions. In this context, the early stratification of patients’ risk for high cumulative hospitalization costs over a longer period (e.g., three years), based on routine information available at their index admission, would be valuable. Specifically, accurate prediction enables hospitals to transition from reactive care to proactive management [7]. This approach could support organizational resource planning, such as the allocation of beds and staff, the prioritization of case management, and the advance arrangement of post-discharge resources [8]. It would also help identify individuals likely to require more intensive and continuous care.

Measuring the hospitalization needs of patients with MDs is a key indicator for assessing disease burden and severity [9]. Although the widespread use of electronic health records (EHRs) and significant advances in health information technology have generated rich longitudinal data for cost prediction [10–12], research on predicting psychiatric inpatient costs in China remains limited. The existing studies have several main limitations. First, many rely on annual summaries or static grouping methods [13–15]. These approaches cannot reflect the dynamic changes in long-term hospital use among patients and are of limited practical use for early risk stratification at the time of admission. Second, different MDs (e.g., schizophrenia, mood disorders, anxiety disorders, and organic mental disorders) show substantial differences in care pathways, length of stay, and cost structure [16–18]. If differences between diagnoses and imbalances in data across disorder types are not explicitly addressed in modeling, predictions for less common disorders often become unreliable [19]. Therefore, a more rigorous predictive framework is needed. This framework should align clearly with research goals, available data, and methods for handling variation among patients. It should serve two purposes: to provide a practical risk stratification tool using information available at admission to aid management decisions, and to systematically examine, in retrospect, how long-term hospitalization patterns are related to cumulative costs.

This study proposes a dual-track analytic framework that integrates static risk stratification and dynamic trajectory interpretation. In the primary analysis, a prediction model for three-year cumulative hospitalization costs is trained and internally validated. This model uses baseline characteristics available at the index admission, such as demographic features, diagnosis groups, and comorbidity index. This process ensures the model can be used prospectively and avoids information leakage. The primary diagnosis groups are also treated as a key predictor. To explicitly show how diagnostic heterogeneity and class imbalance affect predictions, the data is split by diagnostic category, and model performance is reported separately for each group. In the secondary analysis, sequence analysis is used to map patients’ hospitalization trajectories. Unsupervised clustering then identifies subgroups of patients with similar patterns of hospital use. The strength of the association between these trajectory patterns and cumulative costs is quantified. Because this clustering method requires complete three-year follow-up data, it is not suitable for making predictions at the time of admission. Instead, its purpose is to reveal the relationship between structural differences in long-term hospitalization patterns and the resulting cost distribution. Together, these two analyses form a complementary research paradigm: one part provides a tool for prospective risk warning, while the other offers a retrospective explanation of underlying patterns.

From a methodological perspective, machine learning has clear advantages over traditional linear regression when dealing with high-dimensional, nonlinear, and interactive data [20–22]. It has shown better performance in various applications, such as predicting medical costs and stratifying disease risk. In this study, several machine learning algorithms (including random forest, gradient boosting machines, XGBoost, and multilayer perceptron) are used to build prediction models. These models are then systematically compared with traditional linear regression. For the analysis of patient trajectories, methods from sequence analysis, which are well established in fields such as bioinformatics [23] and sociology [24], are applied. Each patient’s hospitalization history is encoded as a time series. Differences between these sequences are measured using an optimal matching algorithm, allowing groups of patients with similar hospitalization patterns to be identified. Previous research has already demonstrated that sequence analysis is useful for describing long-term hospitalization use among patients with MDs [23, 25–27].

This study was conducted using electronic medical record data from the Shenyang Mental Health Center, covering the period from 2017 to 2021. It includes adult patients with MDs who had their index admission at the center during this time and follows them for three years. The study has three main aims: (1) to develop and internally validate a risk-stratification model that predicts a patient’s total hospitalization costs over three years, using only information available on the day of index admission; (2) to identify different patterns of hospitalization trajectories using sequence clustering, to describe the dynamic heterogeneity in disease progression, and to measure how strongly these trajectories are linked to total costs; (3) to use SHAP (SHapley Additive exPlanations) analysis to identify factors associated with cost prediction and to examine differences across diagnostic subgroups. This will help guide the future development of more precise, diagnosis-specific prediction models. Overall, this work aims to provide evidence that can support better planning of mental health resources, improved medical insurance payment policies, and more tailored intervention strategies.

## Methods

### Study participants and dataset

This study followed the TRIPOD-AI (Transparent Reporting of a multivariable prediction model for Individual Prognosis or Diagnosis – Artificial Intelligence) guidelines, with the complete checklist provided in Supplementary Fig. 1. A two-tier analytical design was adopted: (1) the primary analysis developed a prospective cost-prediction model using baseline features available at index admission; and (2) the secondary analysis retrospectively examined hospitalization trajectory patterns and their associations with cumulative costs. This design aimed to differentiate predictive models that can be applied for early clinical risk stratification from purely descriptive association analyses.

**Fig. 1:**
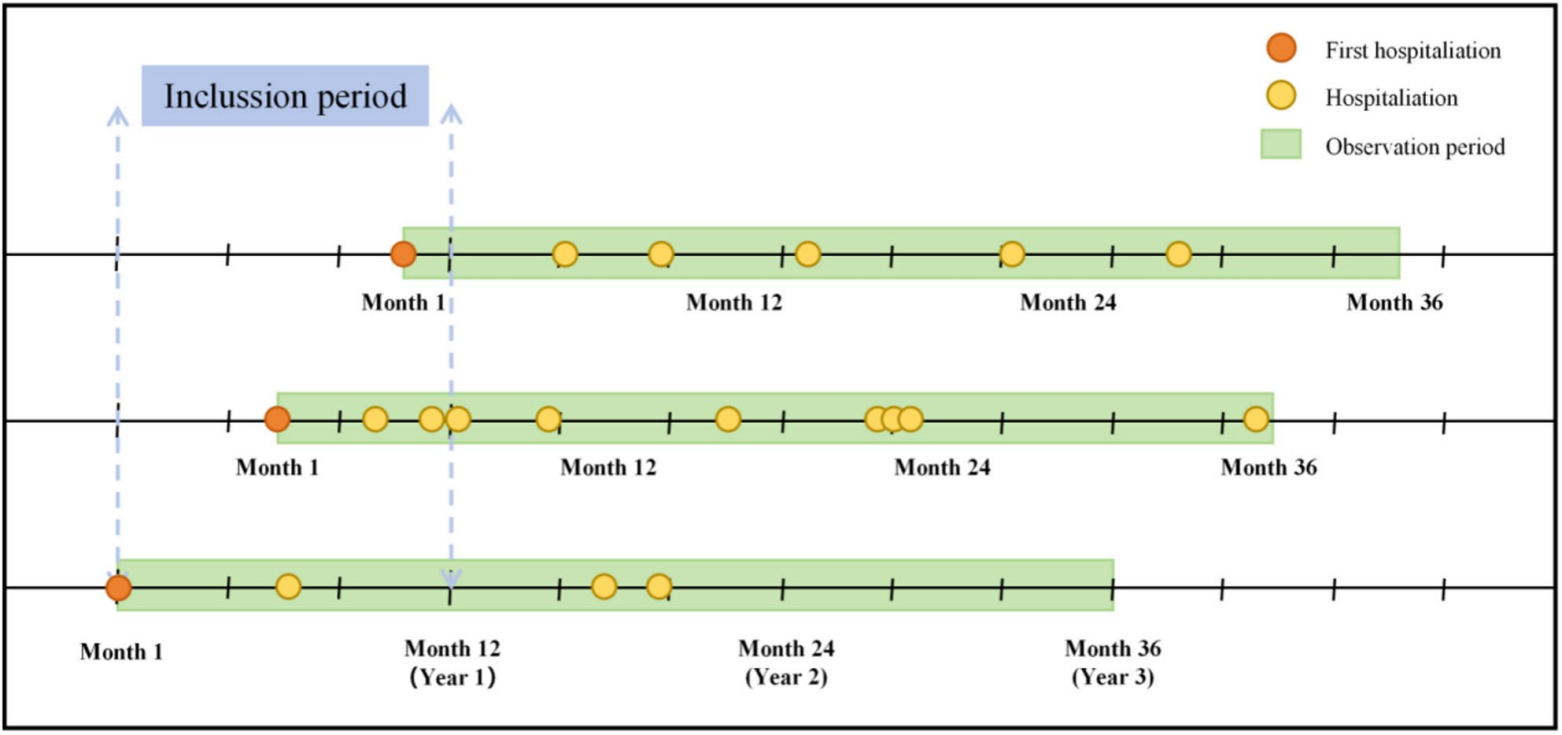
Examples of inclusion criteria for the study subjects. The first hospitalization refers to a patient’s index admission during the inclusion period, which is used as the starting point for observation. From this date, a 1,096-day observation period is constructed. All subsequent hospital admissions within this observation window are referred to as hospitalizations

This retrospective cohort study utilized EHRs data from Shenyang Mental Health Center, China, with the enrollment window restricted to patients who had their index admission between December 16, 2017, and December 16, 2018, allowing for a complete three-year follow-up period through December 2021. Inclusion criteria were: (1) index admission to the Center during this window; (2) primary diagnosis of a mental disorder (ICD-10 codes F00-F99); (3) age ≥ 18 years at index admission; and (4) no in-hospital death during the three-year follow-up period. According to Fig. 1, patients with missing outcome data or any predictor variable with > 20% missingness were excluded. A total of 34 patients (approximately 1.0%) died and were excluded to focus on the cost trajectories of survivors requiring long-term management. For the remaining cohort (missing rate < 5%), missing categorical variables were imputed via mode imputation, while continuous variables were imputed via median. The final dataset comprised 8,896 hospitalization records from 3,396 patients. Based on the events-per-variable (EPV) criterion, the training set (*n* = 2,377) provided approximately 238 EPVs, far exceeding the minimum threshold of 10 EPVs for stable linear model estimation [28], thereby ensuring robustness in parameter estimation. In this study, “index admission” refers to the initial hospitalization at this institution within the study window and may not represent the patient’s first-ever episode of mental disorder, as records from other facilities were unavailable.

This study was approved by the Ethics Committee of Human Experimentation of Shenyang Mental Health Center (reference number: no.2024004). All data were de-identified prior to analysis and stored on password-protected servers with restricted access. No identifiable patient information was disclosed.

### Outcome and predictor variables

*Outcome Variable.* The dependent variable was total hospitalization costs, defined as the sum of all inpatient expenses incurred during the three-year observation period. All costs were standardized using the Chinese Healthcare Consumer Price Index (CPI) and adjusted to 2018 price levels. Given the pronounced right-skewed distribution of hospitalization costs (skewness = 1.13, Supplementary Fig. 2A), a logarithmic transformation [log (cost + 1)] was applied during model training to stabilize variance and reduce the influence of extreme values. The log-transformed distribution is shown in Supplementary Fig. 2B. To correct for retransformation bias when converting predictions back to the original scale, Duan’s smearing estimator was employed, yielding a smearing factor of 1.85.

*Predictor Variables.* Independent variables were extracted from EHRs front-page data and categorized into four groups:

1. Demographic and socioeconomic factors: age, gender, ethnicity, payment method, job and marital status;
2. Disease severity indicators: age-adjusted Charlson Comorbidity Index (aCCI), diagnosis groups (based on the first-level ICD-10), mortality risk level and admission type;
3. Hospitalization-related indicators: length of stay (LOS), discharge status, readmission plan, cost efficiency ratio, and total admissions.

The ten variables in categories (1) and (2) were available at the time of index admission and constituted the primary feature set (Set A) used in the main analysis. Hospitalization-related indicators in category (3), which require follow-up data, were used exclusively for trajectory clustering and sensitivity analyses.

*Variable encoding.* Diagnosis groups were based on the ICD-10 codes of primary diagnoses to their first-level classifications (e.g., F20.0 → F2: Schizophrenia Spectrum Disorders); abbreviated classification names are listed in Supplementary Table 1. The aCCI was calculated as the sum of the original CCI score and an age-based score following the standard definitions of Charlson et al. [29]. Mortality risk was reclassified into three levels: low (0–2 points), moderate (3–4 points), and high (≥ 5 points), reflecting the age distribution and comorbidity patterns specific to this psychiatric population.

### Hospitalization trajectory clustering analysis

Hospitalization trajectory clustering was performed to identify distinct patterns of healthcare utilization and to explore their associations with cumulative costs. Because trajectory clusters can only be determined after the complete three-year observation period, they were not available at the time of index admission and thus cannot be used for prospective prediction. Trajectory cluster membership was therefore included only in the secondary analysis (Set B) to quantify the additional variance in costs explained by utilization patterns (ΔR²); this analysis is explicitly retrospective.

State sequence analysis combined with hierarchical clustering was used to characterize dynamic hospitalization patterns. Each patient’s index admission date was defined as the baseline to establish a fixed 1,096-day observation window (accounting for the leap year 2020). Daily hospitalization status was encoded as a binary sequence (0 = not hospitalized; 1 = hospitalized), producing a 1,096-dimensional time series vector representing the individual hospitalization trajectory.

Using the TraMineR package in R (version 4.4.1), the optimal matching algorithm computed pairwise sequence dissimilarities based on the minimum edit distance required to transform one sequence into another, resulting in an N × N dissimilarity matrix. Hierarchical clustering was then performed using Ward’s minimum variance method. Cluster quality was evaluated using the silhouette coefficient and Calinski–Harabasz index across solutions ranging from k = 2 to k = 8. Considering both statistical criteria and clinical interpretability, a four-cluster solution (k = 4) was selected as optimal. When k = 4, the silhouette coefficient reached its maximum value, indicating both strong intra-cluster compactness and clear inter-cluster separation. Finally, sequence plots were generated for each cluster to visualize daily hospitalization patterns across the 1,096-day observation window.

### Feature selection and preprocessing

To ensure prospective applicability and prevent data leakage, two feature sets were defined:

1. Set A (Primary analysis feature set): Ten baseline variables available at index admission, representing all information accessible to clinicians at patient intake. Models developed using Set A are suitable for early cost-risk stratification.
2. Set B (Secondary analysis feature set): Set A plus the hospitalization trajectory cluster variable. Because cluster membership requires complete three-year observation data, Set B was used exclusively to quantify the association between trajectory patterns and costs (ΔR² = R²(Set B) - R²(Set A)); this analysis has no prospective predictive value.

Data preprocessing was conducted as follows: Categorical variables underwent one-hot encoding with fullRank = TRUE to avoid multicollinearity, producing k-1 dummy variables for each k-category feature. Continuous variables were standardized via Z-score normalization to eliminate scale differences and accelerate model convergence. The outcome variable was log-transformed during training; during evaluation, predictions were back-transformed to the original scale using Duan’s smearing estimator for bias correction. Standardization is particularly important for linear regression and neural network models; although tree-based models (RF, GBM, XGBoost) are relatively insensitive to feature scaling, a unified preprocessing pipeline ensures fair comparison across all modeling approaches. Detailed variable definitions and coding schemes are provided in Supplementary Table 2.

### Data partitioning and cross-validation

All data processing and modeling were conducted at the patient level. Stratified random sampling by primary diagnosis group was applied to preserve diagnostic distributions and minimize sampling bias. Patients were allocated in a 70:30 ratio into a training set (*n* = 2,379) and an independent test set (*n* = 1,017). Within the training set, 5-fold stratified cross-validation was implemented for model training and hyperparameter tuning, ensuring each fold reflected the overall diagnostic composition. For tree-based models (RF, GBM, XGBoost), sample weights were assigned to underrepresented diagnostic categories to mitigate class imbalance effects. To quantify uncertainty in performance estimates, bootstrap resampling $(B=\mathrm{1,000})$ was performed on the test set to derive 95% confidence intervals (CIs) for R², RMSE, and MAE. The CIs were calculated as the 2.5th and 97.5th percentiles of the corresponding bootstrap distributions.

### Model development, training, and evaluation

*Model Selection.* Five prediction models were developed, representing diverse modeling paradigms: ordinary least squares regression (OLS), random forest (RF), gradient boosting machine (GBM), eXtreme gradient boosting (XGBoost), and multilayer perceptron (MLP). OLS serves as the classical linear baseline model and is widely used as a comparative benchmark in international research [30, 31]. RF constructs multiple decision trees via bootstrap aggregation (bagging) and averages their predictions. GBM and XGBoost are both boosting-based ensemble models; however, XGBoost introduces L1/L2 regularization and parallel optimization, significantly enhancing training efficiency and generalizability. The MLP, as a representative deep learning model, captures complex feature interactions through multilayer nonlinear transformations.

*Training and Hyperparameter Tuning.* All models were trained and tuned via stratified five-fold cross-validation within the training set, with log-scale RMSE as the optimization target. Once optimal configurations were identified, models were retrained on the full training set. Hyperparameter search spaces and final configurations are detailed in Supplementary Table 3.

*Performance Evaluation.* Model performance was assessed on the independent test set. Metrics were reported on the log scale. The key evaluation metrics included: R-squared (R²), which represents the proportion of cost variance explained by the model; the root mean squared error (RMSE), which measures the magnitude of the differences between the predicted and actual values with greater sensitivity to large errors; and the mean absolute error (MAE), which intuitively reflects the average absolute magnitude of the prediction errors. All metrics are presented with bootstrap 95% CIs. Pairwise differences in predictive accuracy were evaluated using the Diebold-Mariano (DM) test at α = 0.05. Given heterogeneity in cost structures across diagnostic categories, model performance was additionally reported by diagnosis groups (F0–F4) in a secondary analysis to identify subpopulations with lower predictive accuracy.

*Model Interpretation.* To identify key cost drivers, SHAP analysis [33] was applied to the best-performing nonlinear model. SHAP values quantify each feature’s contribution to individual predictions and reflect associative (not causal) relationships; a high SHAP value indicates predictive importance but does not imply that modifying the feature would alter actual costs. For GBM models trained via the caret package, SHAP values were computed using the iml package. To reduce computational burden, a random subset of 200 test-set samples served as the background dataset. Results were presented as: (1) global feature importance rankings based on mean |SHAP|, aggregated by original variable; (2) SHAP summary plots displaying value distributions and feature-effect relationships; and (3) diagnosis-specific comparisons to explore heterogeneity in cost drivers across conditions.

### Sensitivity analysis

To evaluate the robustness of the primary findings under varying analytical conditions, several sensitivity analyses were conducted. First, a diagnostic subgroup analysis restricted the sample to patients with schizophrenia spectrum disorders (F2) to assess model performance within a single, high-prevalence diagnostic category. Second, patients with cumulative three-year costs exceeding the 95th percentile were excluded to examine the influence of extreme values on model stability. Third, the main analysis was repeated using alternative train-test split ratios (80:20 and 60:40) to explore the effect of training sample size on generalization performance. Finally, two weighting strategies were compared to address diagnostic class imbalance: one applying inverse-frequency weights during training only (with unweighted evaluation), and another applying weights during both training and evaluation. All sensitivity analyses followed the same modeling framework and evaluation metrics (R², RMSE, MAE) as the main analysis.

## Results

### Baseline cohort characteristics

**Fig. 2:**
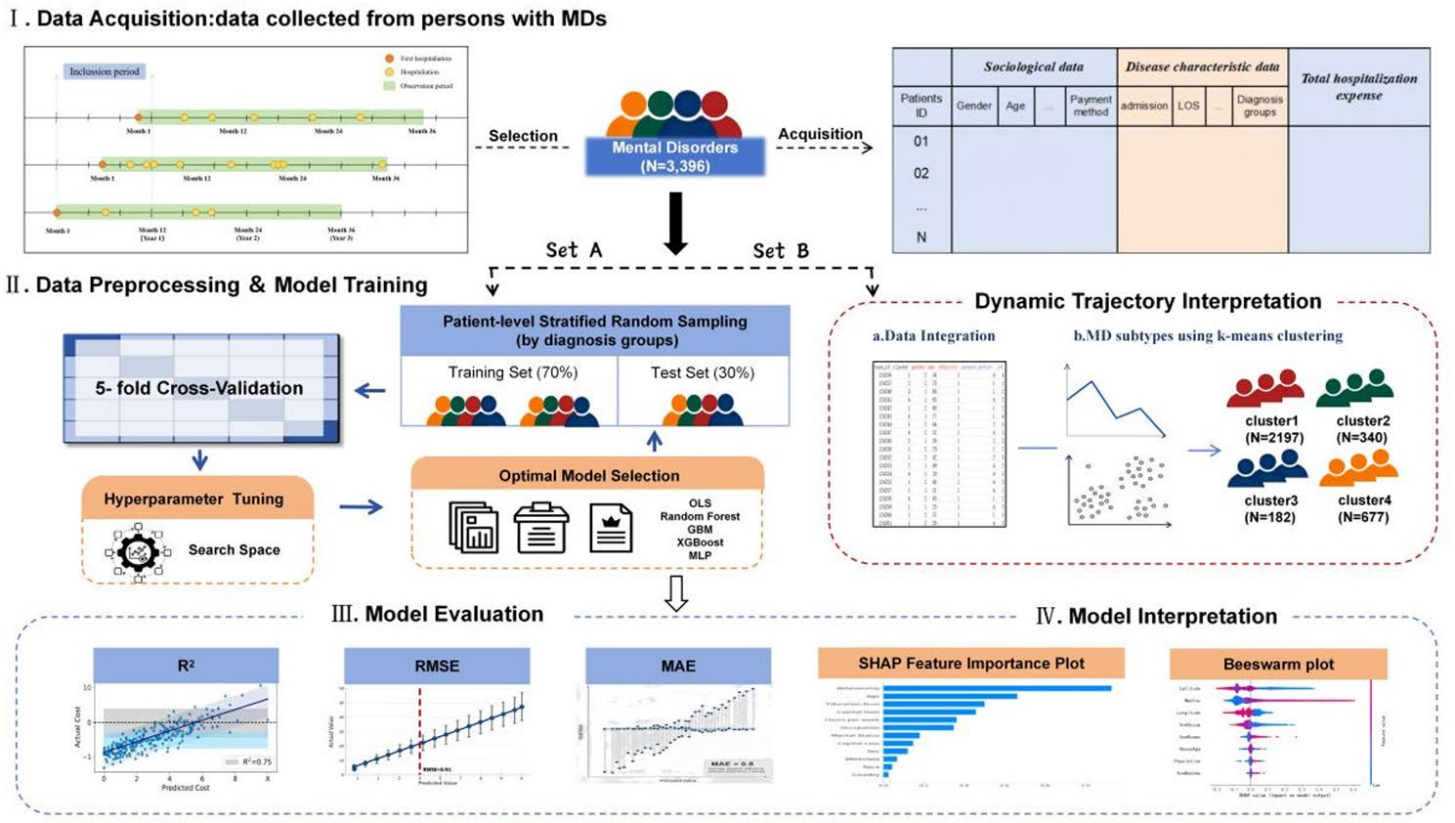
Overall framework of the study design

The overall analytical framework of this study is illustrated in Fig. 2, encompassing four core stages: data acquisition, trajectory clustering, model training, and performance evaluation. Table 1 summarizes the demographic characteristics, clinical features, and hospitalization cost distributions of the study cohort. Overall, patients had a mean age of 47 years, with females comprising a slight majority (54.2%) and Han Chinese ethnicity being predominant (93.7%). Over 40% of patients were unmarried (45.3%) and unemployed (42.0%), with more than half covered by Urban Employee Basic Medical Insurance (UEBMI, 53.9%). Clinically, F2 was the primary diagnosis (61.8%). The aCCI indicated that 35.9% of patients had no comorbidities $(\mathrm{a}\mathrm{C}\mathrm{C}\mathrm{I}=0)$, but patients with a high comorbidity burden (aCCI>1, 35.1%) incurred significantly higher costs (*P* < 0.001). In terms of mortality risk, 84.5% of patients were at low risk, and only 1.3% were at high risk; however, the cost for high-risk patients was 9.6 times that for low-risk patients. Hospitalization cost analysis revealed a pronounced right-skewed distribution. The median three-year cumulative hospitalization cost was ¥35,826 (IQR: 12,084–159,097), with a median cumulative length of stay of 127 days (IQR: 41–565). Payment method, job, aCCI, and diagnosis groups were significantly associated with costs (all *P* < 0.001). The Spearman correlation matrix for all variables is presented in Supplementary Fig. 3.

**Table 1:** Information of patients with MDs

Variable		*N*(%) / (min-max)	Hospitalization costs (Yuan) M (P25,P75)	Z/H value
Gender	Male	1554(45.76%)	35117.05 (11406.03, 180501.58)	0.308
	Female	1842(54.24%)	36520.12 (12469.24, 147698.82)	
Ethnicity	Han Chinese	3182(93.7%)	37971.41 (12564.96, 167940.29)	52.82***
	Manchu	113(3.33%)	13795.38 (6031.66, 35688.12)	
	Hui	34(1%)	28190.99 (12509.83, 142872.73)	
	Other	67(1.97%)	20038.34 (8174.17, 52101.17)	
Payment methods	UEBMI	1831(53.92%)	93344.99 (24511.36, 287244.12)	874.18***
	URRBMI	763(22.47%)	35106.74 (14854.15, 103659.18)	
	RCMS	240 (7.07%)	11147.76 (5829.14, 19546.81)	
	Out-of-Pocket	528 (15.55%)	10173.72 (4859.27, 19007.51)	
	Other	34(1%)	52841.65 (15135.88, 96084.12)	
Job	Employed	511(15.05%)	25912.09 (9676.47, 121405.24)	147.25***
	Retired	561(16.52%)	120566.2 (22473.61, 320530.77)	
	Unemployed	1426(41.99%)	30049.72 (11148.13, 108440.46)	
	Other	898(26.44%)	36219.47 (12045.81, 155605.76)	
Marital status	Unmarried	1537(45.26%)	35124.05 (12858.61, 145035.56)	138.67***
	Married	1121(33.01%)	25352.19 (9345.36, 97387.75)	
	Divorced	479(14.1%)	73664.48 (16285.74, 283123.92)	
	Widowed	146(4.3%)	184775.12 (26093.02, 327860.52)	
	Other	113(3.33%)	83981.7 (17156.29, 280509.75)	
aCCI	0	1220(35.92%)	19496.14 (8779.72, 48843.29)	399.24***
	1	985(29%)	41700.72 (13520.14, 151034.72)	
	> 1	1191(35.07%)	110872.77 (19885.7, 315727.7)	
Diagnosis groups	F0	188(5.54%)	28080.13 (10104.23, 159288.27)	204.14***
	F1	163(4.8%)	15744.97 (5997.14, 44313.91)	
	F2	2097(61.75%)	52613.24 (15908.83, 236491.85)	
	F3	649(19.11%)	23124.16 (9849.24, 78486.82)	
	F4	242(7.13%)	19330.97 (7288.77, 62965.61)	
	Other Diagnosis	57(1.68%)	33464.78 (7472.47, 279207.84)	
Mortality risk level	Low	2870(84.51%)	31111.3 (11312.17, 124389)	158.8***
	Mid	481(14.16%)	131028.89 (24717.03, 321147.43)	
	High	45(1.33%)	295955.25 (26569.37, 357372.44)	
Admit type	Emergency	56(1.65%)	19429.39 (8618.99, 52539.81)	22.86***
	Outpatient	3321(97.79%)	36237.89 (12087.25, 161421.83)	
	Other	19(0.56%)	156596.02 (53905.4, 318503.7)	
Total hospital admissions	1	1657(48.79%)	12427.25 (6065.92, 23941.61)	2307.73***
	2–5	1225(36.07%)	94735.46 (43804.45, 172734.04)	
	> 5	514(15.14%)	324789.5 (295941.38, 339643.74)	
Readmission plan	Yes	3361(98.97%)	36638.43 (12272.16, 161245.76)	5.93***
	No	35(1.03%)	8556.47 (2551.52, 19400.76)	
Discharge status	Cured	254(7.48%)	14329.34 (4041.83, 48029.02)	108.7***
	Improved	3113(91.67%)	38660.03 (13031.08, 169947.98)	
	Not Improved	17(0.5%)	9836.03 (4667.7, 14877.46)	
	Other	12(0.35%)	147553.05 (97786.87, 191502.77)	
Age		18–93	47 (34, 57)	
LOS		1-1096	127 (41, 565.25)	
Cost efficiency ratio		23.54-1999.37	294.38 (266.76, 320.07)	
Total hospitalization costs		24.2-446632.1	35826.3 (12084.06, 159097.01)	

Note: UEBMI: Urban Employee Basic Medical Insurance; URRBMI: Urban and Rural Residents Basic Medical Insurance; RCMS: Rural Cooperative Medical System; aCCI: aged-Charlson Comorbidity Index; LOS: Length of Stay

Z: Mann–Whitney U test (two groups); H: Kruskal–Wallis test (three or more groups); ***: *p* < 0.001

### Hospitalization trajectory clustering

Based on the 1,096-day hospitalization status sequences, state sequence analysis combined with hierarchical clustering identified four distinct hospitalization trajectory patterns (Supplementary Fig. 4). Evaluation using the silhouette coefficient and Calinski-Harabasz index supported the four-cluster solution as optimal (Supplementary Fig. 5). The four clusters were characterized as follows:

#### Cluster 1 (Low-frequency short-stay; *n* = 2,197, 64.7%)

Patients in this cluster had the fewest hospitalization days, with sequence plots dominated by non-hospitalized states (purple areas) and only occasional short-term admissions. This group had the lowest median cumulative cost (¥16,688).

#### Cluster 2 (High-frequency short-stay; *n* = 340, 10.0%)

Patients exhibited high hospitalization frequency but short individual admission durations, with sequence plots showing dense but intermittent short green bands. The median cumulative cost was ¥100,877, and the proportion of F2 diagnoses was relatively high.

#### Cluster 3 (Long-term intermittent; *n* = 162, 4.8%)

Patients exhibited a recurrent pattern of admission–discharge cycles, with alternating green and purple areas suggesting high recurrence rates but moderate single hospitalization duration. The median cumulative cost was ¥183,673.

#### Cluster 4 (Long-term continuous; *n* = 697, 20.5%)

Patients were hospitalized continuously over extended periods, with sequence plots almost entirely filled with green, indicating high-intensity inpatient management needs. This group had the highest median cumulative cost (¥323,549) and median cumulative length of stay (1,096 days), with F2 diagnoses accounting for 81.1%.

Significant differences in demographic and clinical characteristics were observed across the four clusters (Supplementary Tables 4 and 5). Patients in the long-term continuous cluster were older, had more comorbidities, and had higher UEBMI coverage rates, whereas those in the low-frequency short-stay cluster were younger and had higher employment rates. These differences reflect the substantial heterogeneity in disease progression and healthcare service utilization among patients with MDs.

### Model performance evaluation

#### Model performance comparison

Table 2 presents the performance of five prediction models based on the ten baseline variables (Set A) in both the training and independent test sets, with all metrics calculated on the log-transformed cost scale. In the test set, GBM and XGBoost achieved the best and comparable performance (both R² = 0.35; GBM: 95% CI: 0.303–0.396; XGBoost: 95% CI: 0.296–0.396), with the Diebold–Mariano test confirming no significant difference between them (DM = − 0.123, *P* > 0.05). GBM significantly outperformed OLS (DM = − 2.05, *P* < 0.05), random forest (DM = − 5.092, *P* < 0.001), and MLP (DM = − 4.848, *P* < 0.001). Overfitting assessment revealed that although random forest achieved the highest training set performance (R² = 0.528), its test set performance dropped substantially (R² = 0.313, ΔR² = 0.215), indicating severe overfitting. Considering both test set performance and generalization stability, GBM was selected as the optimal model for subsequent analyses.

**Table 2:** Performance evaluation of predictive models for hospitalization costs

Dataset	Model	*R* ^2^	RMSE	MAE	Diebold-Mariano Test
Training set (*N* = 2,377)	OLS	0.361 (0.328–0.391)	1.269 (1.223–1.314)	1.005 (0.973–1.035)	-17.635***
	**Random Forest**	**0.528 (0.503–0.55)**	**1.091 (1.053–1.132)**	**0.855 (0.828–0.885)**	**(Reference)**
	GBM	0.418 (0.391–0.444)	1.211 (1.17–1.253)	0.953 (0.924–0.983)	-19.173***
	XGBoost	0.435 (0.406–0.463)	1.193 (1.153–1.236)	0.938 (0.909–0.967)	-15.992***
	MLP	0.449 (0.418–0.477)	1.178 (1.137–1.218)	0.918 (0.891–0.948)	-10.847***
Test set (*N* = 1,019)	OLS	0.333 (0.283–0.38)	1.258 (1.209–1.309)	1.021 (0.978–1.066)	-2.05*
	Random Forest	0.313 (0.258–0.359)	1.278 (1.221–1.335)	1.031 (0.986–1.079)	-5.092***
	**GBM**	**0.35 (0.303–0.396)**	**1.242 (1.189–1.293)**	**1.005 (0.96–1.049)**	**(Reference)**
	XGBoost	0.35 (0.296–0.396)	1.243 (1.191–1.296)	1.001 (0.958–1.047)	-0.123
	MLP	0.274 (0.212–0.331)	1.313 (1.254–1.374)	1.046 (0.997–1.096)	-4.848***

Note: All metrics are calculated on the log-transformed cost scale (log (Cost + 1)). R² = coefficient of determination; RMSE = root mean squared error; MAE = mean absolute error. Values in parentheses represent 95% confidence intervals (1,000 iterations); Diebold-Mariano Test: Negative values indicate the comparator model performs significantly worse than the reference model (RandomForest for training, GBM for test). *: *p* < 0.05;***p < 0.001. Bold values indicate best-performing model in each dataset

#### Model calibration and residual diagnostics

**Fig. 3:**
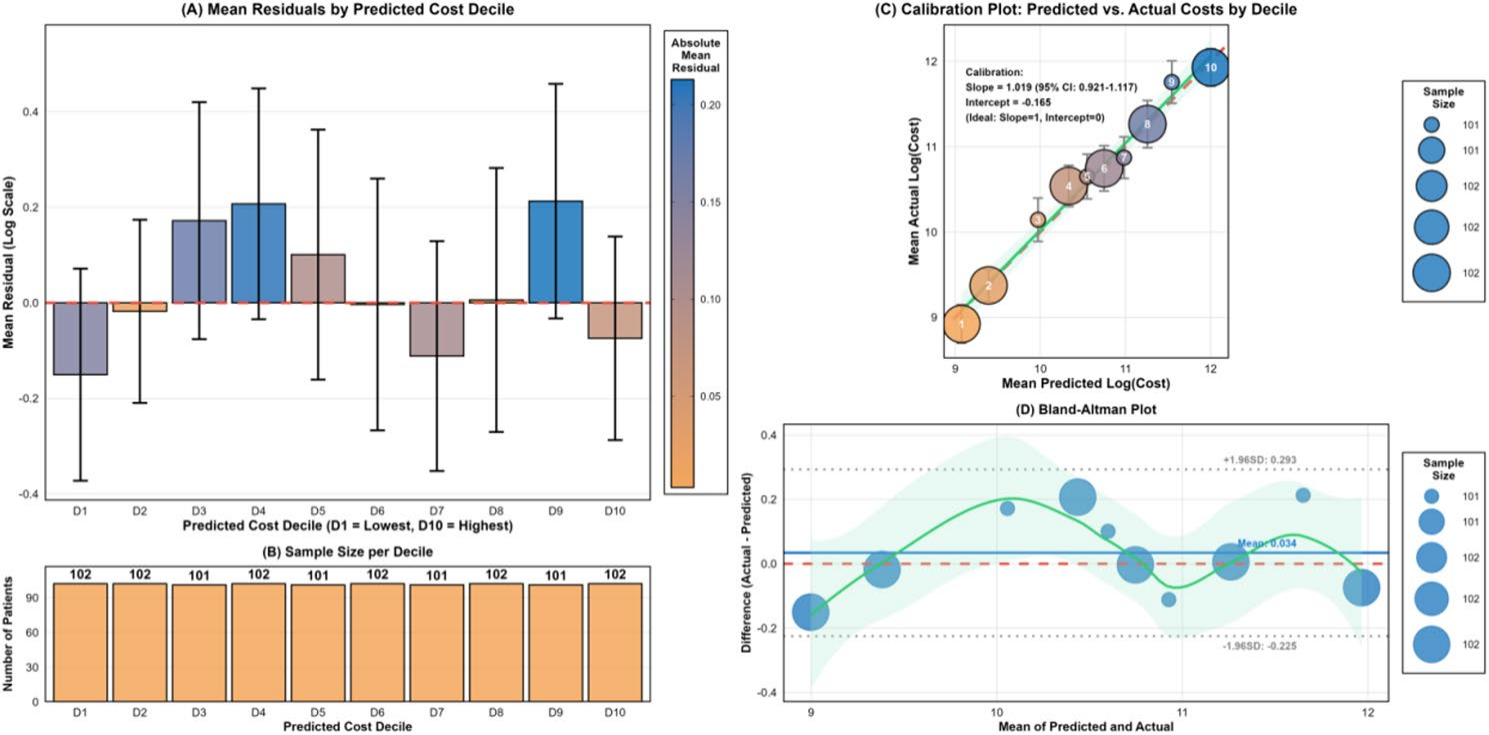
Comprehensive calibration analysis. This figure evaluates the predictive performance of the GBM model from four aspects: residual distribution, sample size, calibration curve, and agreement limits. (**A**) Mean Residuals by Predicted Cost Decile. If the residuals are symmetrically distributed around zero and all confidence intervals cross zero, it indicates that the model has no significant systematic bias. (**B**) Sample size per Decile. This shows the number of samples in each predicted cost decile group, confirming that each group has sufficient and balanced sample sizes. (**C**) Calibration Plot. This scatter plot shows the mean predicted cost versus the mean actual cost for each decile, with the ideal diagonal line (y = x) added. The closer the points are to the diagonal line, the better the calibration. (**D**) Bland-Altman plot. This plot shows the error distribution at the individual level, with the mean of predictions on the x-axis and the prediction error (residual) on the y-axis. If most points fall within the ± 1.96 standard deviation limits and show no obvious trend, it indicates good agreement in model predictions

To assess model reliability, comprehensive calibration analysis was conducted for the GBM model. Residual analysis by predicted cost decile revealed no systematic bias, with 95% confidence intervals crossing zero for all ten deciles (Fig. 3A-B). Mean residuals ranged from − 0.13 to + 0.22 log units across deciles, indicating balanced prediction accuracy across the full cost spectrum without systematic bias. Calibration analysis demonstrated good agreement between predicted and actual values (Fig. 3C), with a calibration slope of 1.019 (95% CI: 0.921–1.117), close to the ideal value of 1.0, and an intercept of − 0.165, close to zero. All decile points clustered tightly around the identity line. Bland–Altman analysis (Fig. 3D) further confirmed minimal bias, with all observations falling within the ± 1.96 SD limits of agreement (upper limit: 0.293; lower limit: −0.225). Detailed residual diagnostics (Supplementary Fig. 6) showed that the LOESS curve remained close to zero across the full range of predicted values, and the Q-Q plot indicated that the residual distribution approximated normality, with only slight deviations in the extreme tails. Collectively, these calibration and residual diagnostics demonstrate that the GBM model provides well-calibrated predictions across the entire range of hospitalization costs, supporting its reliability for subsequent SHAP analysis to identify cost drivers.

#### Model performance by diagnosis group

**Fig. 4:**
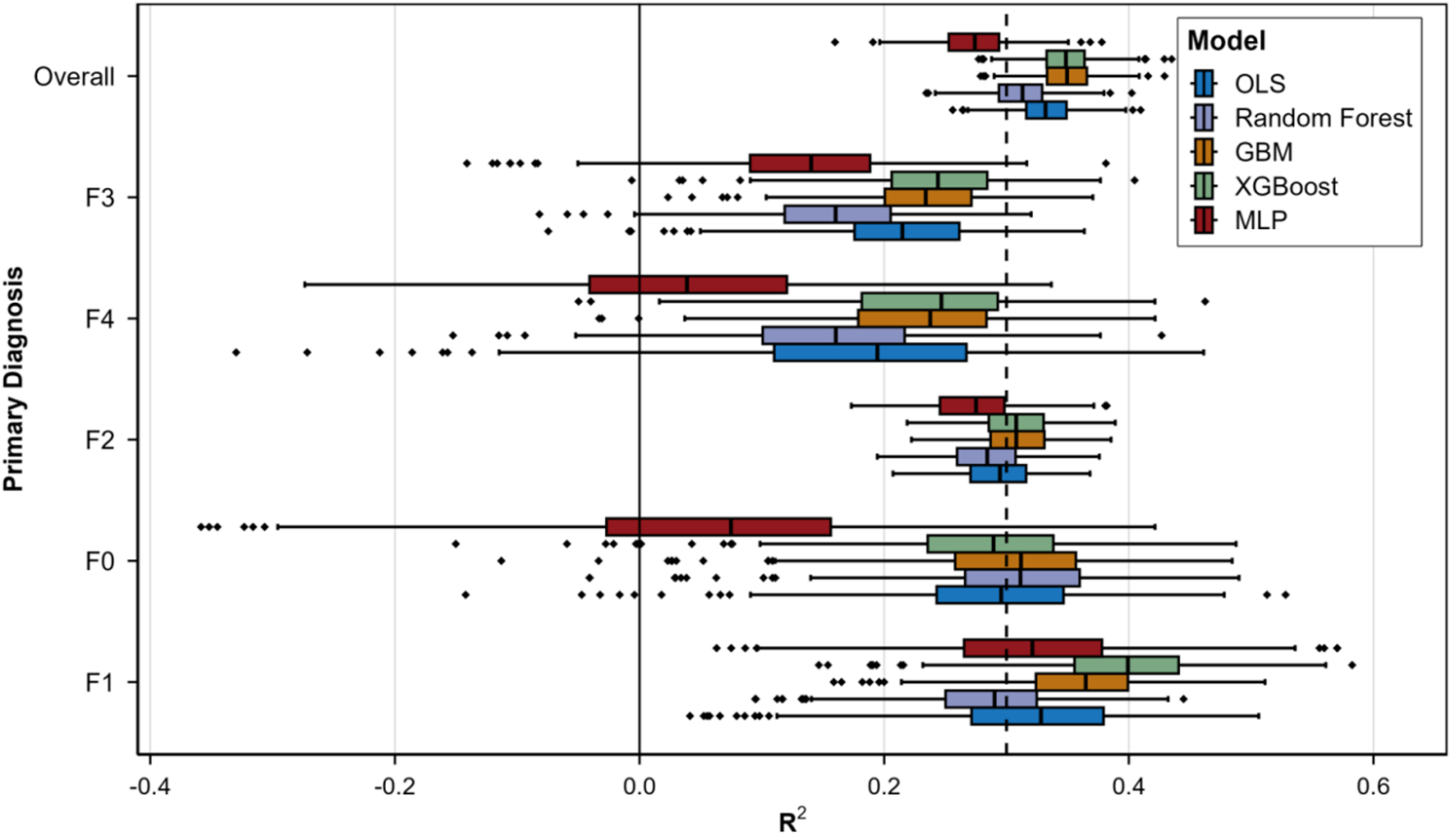
Model Performance by diagnosis groups. Box plot of R^2^ values evaluated per primary diagnosis group. Five predictive models (OLS, Random Forest, GBM, XGBoost, MLP) were trained on the full dataset and evaluated on diagnosis-specific test subsets. Box elements: thick line = median; box limits = Q1 and Q3; whiskers = Q1-1.5IQR to Q3 + 1.5IQR; points = outliers. Vertical lines: solid (R²=0) and dashed (R²=0.3). Diagnosis groups ordered by descending GBM median R²

Given the heterogeneity in cost structures and service utilization across different mental disorder categories, Fig. 4 presents the prediction performance of all five models stratified by diagnosis group. Models were trained on the full dataset and evaluated on diagnosis-specific test subsets. The F2 subgroup (comprising 61.8% of the sample) exhibited the most stable model performance, with GBM achieving a median R² of approximately 0.35, comparable to the overall performance. Performance in the F3 and F4 subgroups was slightly lower but remained acceptable. In contrast, the F0 and F1 subgroups showed substantially poorer and more variable model performance due to limited sample sizes (approximately 5% and 3%, respectively), with MLP exhibiting particularly unstable performance in the F0 subgroup. These results indicate that the current model demonstrates strong predictive capability for the predominant diagnosis group (F2) but limited precision for minority diagnostic categories (F0, F1). Future research may consider developing diagnosis-specific models or utilizing multi-center data to increase sample sizes for minority classes and improve prediction performance.

### Secondary analysis: association between trajectory patterns and costs (Set B)

To quantify the explanatory power of hospitalization trajectory patterns on cost variation, trajectory cluster membership was added to Set A to form Set B, and a retrospective association analysis was conducted using the GBM model (Supplementary Table 6).

The Set B model achieved a test set R² of 0.712 (95% CI: 0.680–0.741), representing a substantial improvement over Set A (R² = 0.35). The additional explanatory power attributable to trajectory clustering was: ΔR^2^ = 0.362. This indicates that hospitalization trajectory patterns explained an additional 36.2% of the variance in costs. However, because trajectory clustering requires complete three-year observation data, the Set B model has no prospective predictive value. It reflects only the strength of association between trajectory patterns and costs—that is, patients with different hospitalization patterns exhibit significant systematic differences in three-year cumulative costs—rather than incremental information available for prediction at the time of index admission. This finding suggests that hospitalization trajectory is an important explanatory factor for costs, and future research may explore predictive indicators that can identify patients at high risk for intensive hospitalization trajectories early, thereby enabling earlier intervention.

### SHAP-based model interpretation

SHAP analysis based on the GBM model (Set A) elucidated the contribution patterns of features to hospitalization cost prediction. It is important to note that SHAP values reflect the marginal contribution of features to the prediction result, quantifying associative rather than causal relationships.

#### Global feature importance

**Fig. 5:**
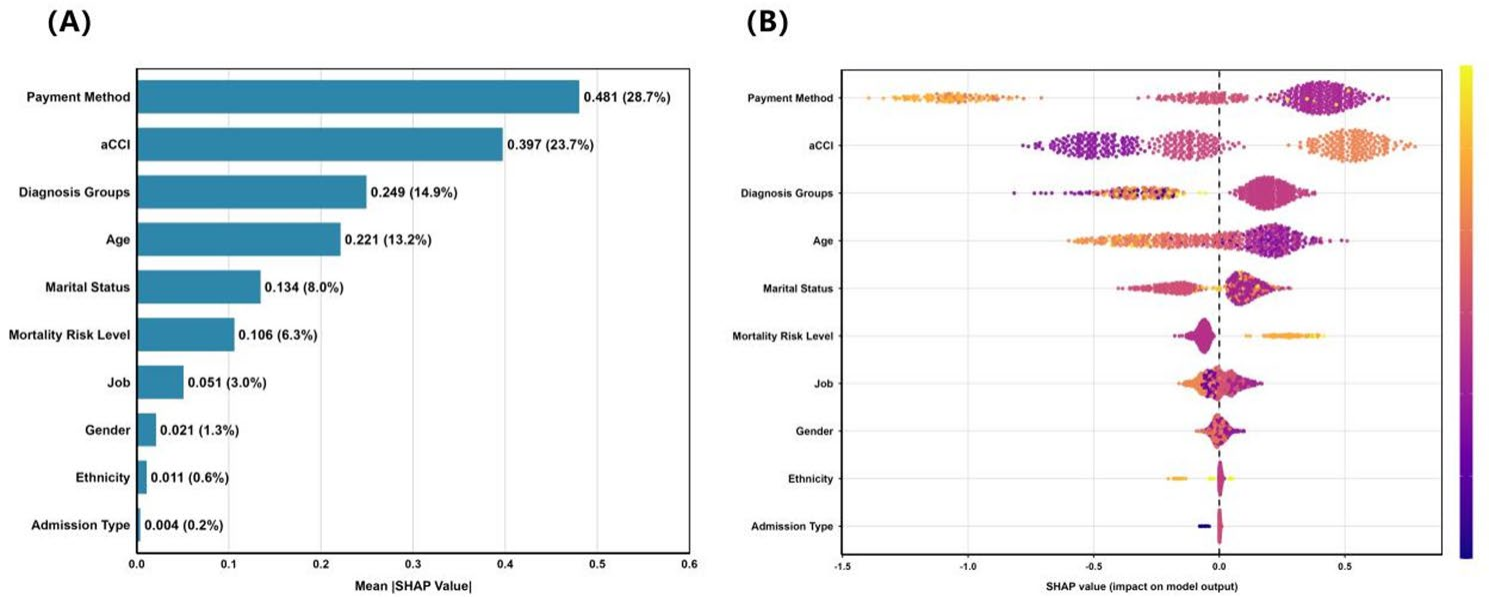
Interpretation of the GBM model. (**A**) Importance ranking of the 10 identified features according to the mean (|SHAP value|). (**B**) Summary plot based on the SHAP values. A higher SHAP value of a feature suggests a higher risk contribution. Colors on the plot denote the magnitude of the feature values, wherein high values are indicated in yellow, while low values are shown in purple

Figure 5A illustrates how much each baseline feature contributed to cost predictions. Payment method emerged as the predominant predictor (mean |SHAP| = 0.481, 28.7%). The next most important features were the aCCI (0.397, 23.7%), diagnosis groups (0.249, 14.9%), and age (0.221, 13.2%). Together, these four features accounted for over 80% of the model’s predictions. They were much more important than demographic factors like gender (1.3%) and ethnicity (0.6%).

#### Directional feature impact

Figure 5B shows how feature values affect predicted costs. A distinct positive correlation was observed between costs and both comorbidity burden and age. Higher values led to positive SHAP values, which means higher predicted costs. Patients with high mortality risk also showed a strong increase in predicted costs. For diagnosis groups and payment methods, different categories had different effects. For example, certain payment methods and the F2 diagnosis group increased the predicted costs, while others decreased them.

#### Feature importance by diagnosis group

Supplementary Fig. 7 shows that the importance of cost drivers varies across diagnosis group. Payment method was the top predictor for all subgroups (F0-F4), but its influence was strongest in the F4 group (mean |SHAP| = 0.541) and weakest in the F1 group (0.385). For the F0 group, the aCCI (0.444) was almost as important as the payment method (0.450), and age (0.317) was a much stronger predictor than in other groups. The F2 group showed a pattern very similar to the overall population. The top three drivers were payment method, aCCI, and age. Marital status was a more important predictor for F2 and F3 patients compared to F0 or F1 patients.

### Sensitivity analyses

To evaluate the robustness of the primary findings, model training and evaluation were repeated under varying sample selection criteria, data partitioning ratios, and weighting strategies (Supplementary Table 7). When the analysis was restricted to F2 patients (*n* = 2,097), the GBM model achieved a test set R² of 0.304 (95% CI: 0.239–0.365), slightly lower than the main analysis, suggesting that although diagnostic homogenization reduced heterogeneity, the reduced sample size (from 3,396 to 2,097) may have limited model learning capacity. After excluding 170 patients with costs exceeding the 95th percentile, the model R² was 0.312 (95% CI: 0.264–0.358), similar to the main analysis, indicating that extreme high-cost patients had limited impact on overall prediction performance. When alternative train–test split ratios were applied, the 80:20 split yielded an R² of 0.392 (95% CI: 0.336–0.441), significantly higher than the main analysis, likely attributable to the larger training set (*n* = 2,717 vs. 2,380) enhancing model fitting capacity; the 60:40 split yielded an R² of 0.349 (95% CI: 0.305–0.394), essentially equivalent to the main analysis, suggesting that the 70:30 split achieved a reasonable balance between training efficiency and evaluation reliability. Regarding weighting strategies, training-only weighting yielded an R² of 0.315 (95% CI: 0.268–0.358), and weighting during both training and evaluation yielded an R² of 0.307 (95% CI: 0.197–0.409); both were slightly lower than the main analysis with wider confidence intervals, indicating that although weighting strategies may improve performance for minority diagnosis groups, they may reduce overall prediction stability. In summary, sensitivity analyses demonstrated that the main analysis conclusions are robust, with model performance remaining relatively stable across variations in sample selection and data partitioning strategies. The performance improvement observed with the 80:20 split suggests that increasing training sample size may further enhance prediction accuracy.

## Discussion

### Main findings

This study provides preliminary evidence that risk stratification for three-year cumulative hospitalization costs is feasible using only routine baseline information collected from electronic medical records at a patient’s first admission to a psychiatric hospital. Under strict conditions regarding the timing of variable collection and the avoidance of information leakage, a gradient boosting machine model was developed using ten baseline variables available on the day of first admission. On the independent test set, the model achieved an R² value of 0.35 (95% CI: 0.303–0.396) for log-transformed hospitalization costs, explaining approximately one-third of the variance in long-term inpatient costs. Calibration analysis showed good agreement between predicted and observed costs, with a calibration slope of 1.019. Residual analysis revealed no evidence of systematic bias, indicating stable predictive performance across the full range of cost values. Although the current model accuracy is not yet sufficient for precise individual-level cost prediction, it can serve as a screening tool for early identification of patients at high risk of high resource use [16], offering data-driven support for institutional management decisions and resource allocation.

A core contribution of this study is the development and clear differentiation of a dual-track complementary framework that separates prediction from explanation. The prediction track (Set A) relies solely on information available at the time of first admission, including demographic characteristics, payment methods, and aCCI, to enable prospective risk stratification and early risk alerts for newly admitted patients. The explanation track (Set B), in contrast, uses hospitalization trajectory clusters derived from complete three-year follow-up data via sequence clustering. This track is used exclusively for retrospective description and outcome analysis, aiming to clarify why some patients incur substantially higher hospitalization costs. After adding the trajectory cluster variable to the model, the explained variance increased to approximately R² = 0.71, representing a gain of about 0.36 in explanatory power. This substantial increase indicates a strong association between long-term inpatient service utilization patterns and cumulative costs [32]; however, this explanatory component cannot be used for prediction at the time of admission.

This analytical framework clarifies the mechanism underlying long-term hospitalization costs for patients with MDs. A patient’s baseline characteristics determine their initial risk level for incurring high costs. Subsequent dynamic trajectories of inpatient service utilization, such as prolonged or frequent readmissions, are shaped by multiple factors, including disease progression and the health care delivery system, and serve as key drivers of additional cost accumulation. Therefore, accurately distinguishing the functional and temporal differences between prediction and explanation is essential for correctly interpreting and applying the findings of this study. Such a distinction not only prevents the misuse of retrospective variables in prospective prediction tasks but also offers a clear methodological reference for future research on long-term cost prediction in mental health settings [18, 33].

### Interpretation of hospitalization trajectory types

This study used state sequence analysis combined with hierarchical clustering to identify four distinct trajectory patterns based on three-year hospitalization data. The statistical validity of the four-cluster solution was supported by multiple indices. Supplementary Fig. 5 shows the silhouette coefficient and Calinski–Harabasz index for different numbers of clusters (k = 2 to 8). At k = 4, these indices either reached their peak values or entered a stable plateau, indicating satisfactory intra-cluster cohesion and inter-cluster separation. This suggests that the identified cluster structure is unlikely to have arisen from random noise alone [34, 35].

The four trajectory types differed significantly in demographic characteristics, baseline clinical profiles, length of stay, and cumulative costs. These differences indicate that the clustering solution is not only statistically meaningful but also corresponds to patient subgroups with distinct patterns of resource utilization. The low-frequency, short-stay type accounted for 64.7% of the sample. Patients in this group were generally younger and had higher employment rates. The median three-year cumulative cost was ¥ 16,688, the lowest among all four groups. This trajectory represents the majority of patients with relatively low hospitalization needs. The high-frequency, short-stay type comprised 10.0% of the sample and was characterized by repeated short-term hospitalizations. Their costs fell within a moderate range. The long-term, intermittent type accounted for 4.8% of patients. These individuals experienced longer hospital stays but with clear intervals between admissions. Their costs were also at a moderate level. The long-term, persistent type represented 20.5% of the sample. Patients in this group were older, had higher comorbidity burdens, and were more likely to be covered by UEBMI. The proportion of patients with an F2 diagnosis reached 81.1%. The median three-year cumulative cost for this group was ¥ 323,549. This cluster clearly reflects a high-need, high-cost population.

These trajectory types are comparable to service utilization typologies reported in international studies [36, 37]. The long-term persistent type closely resembles patterns described as prolonged hospitalization or institutionalized care trajectories [26]. The high-frequency short-stay type is highly consistent with the characteristics of the “revolving door” population frequently discussed in the literature [38]. Other trajectory patterns also have counterparts commonly observed in prior sequence analysis studies in psychiatry [24]. This cross-study consistency provides preliminary support for the external validity of our findings.

It is important to emphasize that the trajectory clusters identified in this study are data-driven and can only be determined after complete three-year follow-up data are available. Therefore, in this study, trajectory cluster membership is strictly defined as a retrospective descriptive and explanatory tool. Its value lies in helping to answer the question of why certain patient groups incur substantially higher long-term costs—for example, by revealing that high costs are often associated with persistent, long-term hospitalization patterns. It is not intended for prospective prediction at the time of a patient’s first admission. This distinction is a critical methodological premise for interpreting the clinical relevance of trajectory analysis and also defines the boundaries for future efforts aimed at identifying early detectable signals of high-risk trajectories.

### Diagnostic heterogeneity, model performance, and practical application

This study evaluated model performance stratified by diagnostic category, and the results revealed substantial diagnostic heterogeneity. The overall stability of the global model was mainly driven by the F2 group (schizophrenia spectrum disorders), which had the largest sample size and the highest internal homogeneity, accounting for 61.8% of the cohort. The median R² value for this subgroup was approximately 0.35, consistent with the overall model performance. Model performance in the F3 and F4 groups was slightly lower but remained within an acceptable range. In contrast, performance in the F0 and F1 groups decreased markedly, with considerably wider confidence intervals.

These differences reflect fundamental distinctions in how inpatient costs are generated across diagnostic categories. For F2 patients, the disease course is relatively predictable, characterized by chronicity and recurrent episodes. For F0 patients, hospitalization costs are more strongly influenced by the progression of somatic comorbidities. For F1 patients, costs are closely related to factors such as mandatory detoxification cycles and social behavioral circumstances. When a global model is applied to a mixed cohort encompassing multiple diagnostic categories, it is difficult to simultaneously capture the heterogeneous drivers of costs specific to each diagnosis.

SHAP analysis provided further evidence of diagnosis-specific patterns in the model. Payment method was the most important predictor across all diagnostic subgroups, but its relative influence varied. The strongest effect was observed in the F4 group, with a mean absolute SHAP value of 0.541, while the weakest effect was in the F1 group, with a value of 0.385. In the F0 group, age and aCCI were substantially more important than in other groups, consistent with the clinical characteristics of patients with organic mental disorders, who tend to be older and have more physical comorbidities. Based on these findings, the global model is positioned as a preliminary risk stratification tool for the overall inpatient population in psychiatric institutions. Its scope of applicability is defined by the diagnosis-stratified performance results. Future efforts to improve predictive stability for less common diagnostic categories may consider expanding the sample through multi-center studies, developing diagnosis-specific models, or employing multi-task learning approaches.

In terms of practical application, given the current predictive performance and the availability of input variables, the model is more suitable as an auxiliary tool for institution-level resource planning. Model outputs should be converted into risk stratification categories (e.g., identifying the top 10% or 20% of patients at highest risk) to flag potential high-resource users, rather than being used for precise individual-level cost prediction. This type of output is more aligned with the actual needs of management decision-making and reduces the risk of misinterpretation or misuse of predictions. The key predictors identified through SHAP analysis (e.g., payment method, aCCI, diagnosis groups, and age) can serve as reference points for clinical and administrative teams when assessing patient risk and understanding why a patient is classified as high-risk. However, these factors should not be directly treated as intervention targets or used as the basis for automated decision-making.

At the same time, high-risk patient alerts can be integrated into existing workflows, including multidisciplinary rounds, discharge planning, and health resource coordination. For patients identified as high-risk, prioritized follow-up, case management, and linkage to community-based rehabilitation services can be arranged, thereby translating risk signals into concrete management actions. It should be acknowledged that the current study has only completed feasibility testing of the model and the development of the methodological framework. The operability of the model in real-world clinical settings, its acceptance by health care professionals, and its actual impact on resource allocation efficiency have not yet been examined. A gap exists between model complexity and clinical implementation; translating SHAP-based insights into actionable decision rules requires further simplification and validation. Future research should therefore focus on developing simplified risk scores or decision support tools that can be seamlessly integrated into clinical workflows. Successful implementation of the model will require further resolution of practical issues such as integration with electronic health record systems, standardized data management, staff training, and workflow optimization. Future prospective implementation studies are needed to further validate the practical value and applicable boundaries of the model.

### Policy context and payment system

SHAP analysis showed that payment method was the most important predictor in the model, accounting for 28.7% of the total predictive weight. This finding suggests that different insurance schemes in China (e.g., Urban Employee Basic Medical Insurance [UEBMI] and Urban and Rural Residents Basic Medical Insurance [URRBMI]) vary in reimbursement rates, scope of designated providers, and cost control mechanisms. These differences may indirectly reflect patient characteristics such as socioeconomic status, disease severity, or health care accessibility, which are not fully captured by other baseline variables. The relatively higher reimbursement rates of UEBMI may reduce patients’ out-of-pocket financial burden [32], potentially leading to extended length of stay or increased service intensity even in the absence of clear clinical indications. Whether this reflects supplier-induced demand or moral hazard requires further investigation through health economics research [39].

It is important to clarify that the observed association between payment method and cumulative hospitalization costs is correlational only, and no causal relationship can be established. Payment method should not be interpreted as a direct driver of higher costs, but rather as a proxy for broader contextual factors. Its practical value lies in its availability at admission and its contribution to improving early risk stratification.

From a policy perspective, trajectory analysis revealed that approximately one-quarter of patients exhibited high-intensity hospitalization patterns (frequent short stays or prolonged continuous stays). These patterns suggest a potential misalignment between the long-term care needs of some patients with MDs and the incentives embedded in traditional fee-for-service payment models [40]. Whether alternative payment mechanisms (e.g. case-based payment, capitation, or psychiatric-specific models) could improve efficiency or reduce non-clinically necessary hospitalizations remains an open question. As this study is a single-center retrospective analysis not designed for policy evaluation, such considerations are preliminary and require formal testing through multi-center health economics research or payment reform pilot evaluations.

## Limitations

First, the retrospective study design was used only to test the technical feasibility of cost risk prediction and did not involve causal inference or counterfactual analyses. Therefore, the study findings cannot demonstrate superiority over current clinical management practices. Second, patients who died during the follow-up period were excluded from the analysis. While this exclusion was intended to focus on hospitalization management trajectories among longer-term survivors, it may have introduced some degree of survivor bias. Third, in terms of model development, this study deliberately restricted predictor variables to those available at the time of first admission and did not employ autoregressive model structures. As a result, the R² value of 0.35 represents the predictive performance achievable using only routine baseline information and does not reflect the theoretical upper limit of long-term hospitalization cost prediction. The stability of the model across different policy periods has not been tested and requires further evaluation in prospective cohort studies. Fourth, autoregressive terms or prior cost history were not included as predictors, consistent with our focus on admission-day risk stratification and constrained by the lack of linked inter-institutional data. Future studies should incorporate such benchmarks when longitudinal data become available. These research boundaries and limitations provide a clear reference for appropriately interpreting the study findings and for identifying directions for future research.

## Conclusion

Using electronic medical record data from a tertiary psychiatric hospital in Northeast China, this study provides preliminary evidence that risk stratification for three-year cumulative hospitalization costs is feasible based solely on routine baseline information collected at a patient’s first admission (prediction track, Set A; test set R² ≈ 0.35), offering data support for service-level resource planning. Through retrospective trajectory clustering, four distinct hospitalization utilization patterns were identified and shown to be strongly associated with cumulative costs (explanation track, Set B; R² ≈ 0.71, explanatory gain 0.36), indicating that dynamic inpatient service use is a core driver of long-term cost differences, although trajectory information is only valuable for retrospective explanation and cannot be used for admission-day prediction. These findings demonstrate that machine learning models built on routine clinical data can serve as auxiliary tools in mental health service management, supporting the transition from passive payment models to proactive resource planning. As a single-center retrospective study, predictive performance in certain diagnostic subgroups requires improvement, and generalizability to other settings remains to be tested. Future research should focus on external validation using multi-center data, development of diagnosis-specific models, integration of additional early-admission variables, and prospective implementation studies to evaluate real-world effectiveness and feasibility.

## Supplementary Information

Below is the link to the electronic supplementary material.

Supplementary Material 1

## Data Availability

Researchers interested in replicating the core findings or collaborating under specific research questions may contact the corresponding author to discuss potential access under a formal data use agreement, in full compliance with ethical and legal requirements.

## Code availability

To ensure the replicability of our methodology, we will deposit the analysis code (R/Python scripts) for model training, validation, and visualization on a public code repository (e.g., GitHub) upon manuscript acceptance.

## Abbreviations

MDs: Mental disorders
DALYs: Disability-adjusted life years
YLDs: Years lived with disability
WHO: World Health Organization
EHRs: Electronic health records
ICD-10: International classification of diseases, 10th revision
aCCI: Age-adjusted charlson comorbidity index
CPI: Consumer price index
OLS: Ordinary least squares
RF: Random forest
GBM: Gradient boosting machine
XGBoost: eXtreme gradient boosting
MLP: Multi-layer perceptron
R^2^: R-square
RMSE: Root mean squared error
MAE: Mean absolute error
SHAP: SHapley additive explanations
LOS: Length of stay
UEBMI: Urban employee basic medical insurance
URRBMI: urban‒rural resident medical insurance
UEBMI: Urban employee basic medical insurance
CIs: Confidence intervals
DM: Diebold-mariano test
