# Supplementary material for: Risk stratification for long-term inpatient costs in mental disorders: a dual-track machine learning approach using baseline EHRs and hospitalization trajectories
Source: BMC Health Serv Res. 2026 Feb 28;26:359. doi: 10.1186/s12913-026-14274-y (PMC12988640; doi:10.1186/s12913-026-14274-y)
Supplement: Supplementary file 1 — Supplementary Material 1 [file 12913_2026_14274_MOESM1_ESM.docx]

**Study Setting and Healthcare Context**

**1. Hierarchy, Regional Role, and Service Landscape of the Study Hospital**

In China, mental health services are provided through a standardized hospital tier system. According to the National Health Commission’s “ Hospital Grading Management Measures,” psychiatric specialist hospitals are evaluated under a three-level, six-class rating system. The core criteria for this rating include medical quality, specialist capability, and management standards. Within this system, tertiary Grade A psychiatric hospitals represent the highest level of service capacity and typically function as regional referral centers for complex and severe cases.

Nationally, mental health services exhibit a distinct pattern of being “specialist hospital-dominated.” Although some general hospitals operate psychiatric outpatient departments, inpatient services—especially for patients whose primary diagnosis is a mental disorder—are almost exclusively provided by psychiatric specialist hospitals. This is primarily due to two factors: first, the vast majority of mental health professionals are concentrated in these specialist hospitals; and second, the mental health service capacity of general hospitals is relatively limited. This structural division of services gives unique value to studying the patterns of non-psychiatric (i.e., cross-specialty) service use among psychiatric inpatients.

This study was conducted at the Shenyang Mental Health Center. This public, tertiary Grade A psychiatric hospital exemplifies the typical role of a regional core institution in three key dimensions:

①Scale: It is the largest psychiatric hospital in Liaoning Province, with 1,189 authorized beds and 1,520 operational beds, meeting the national requirements for a tertiary psychiatric hospital.

②Function: The hospital also operates under the titles “Shenyang Center for Mental Disease Prevention and Control” and “Shenyang Mental Health Service Center.” This integrated “clinical care and public health” structure means it not only provides clinical treatment but also coordinates city-wide mental health prevention, crisis intervention, technical guidance, and staff training (managing over 300 mental health workers in the city), serving the entire population across the lifespan.

③Disciplinary Development: The hospital hosts several regional specialist centers (e.g., for child mental health, depression treatment, sleep medicine), reflecting the standard practice of high-level specialist centers in managing a complex spectrum of disorders (“specialized care for specific disorders”).

The core rationale for selecting this center lies in the high representativeness of its patient population. Within China’s tiered healthcare system, patients with severe, complex, or difficult-to-treat mental disorders tend to be referred to such regional tertiary specialist centers. Therefore, analyzing its inpatient patterns most effectively reveals the key characteristics of the patient groups with the highest demand for mental health services and the most significant resource consumption under the current medical and health insurance policies. Furthermore, as a top-tier specialist center and public health management institution, its standardized clinical practices and medical records provide a reliable data foundation. The findings of this study hold important reference value for understanding other Chinese regional mental health centers of a similar level and function.

**2. Guideline-Based Standardized Clinical Practice and Admission Pathways**

The clinical operations at this center strictly follow the national “Evaluation Standards for Tertiary Psychiatric Hospitals” and internal management systems, with the core goals of ensuring patient safety and improving clinical outcomes. Its principles are consistent with mainstream international guidelines (e.g., APA, NICE). The main admission indications comprehensively reference both domestic and international guidelines, including: the presence of a clear and imminent risk of self-harm or harm to others; acute or severe psychotic symptoms or affective episodes causing significant functional impairment; or the need to urgently initiate complex treatment with insufficient community support.

The specific clinical pathway follows a clear, standardized process (Supplementary Figure S1). This process begins with an initial assessment in the outpatient/emergency department. Admission must be reviewed and approved by an attending physician or higher, based on established criteria, before proceeding. Upon admission, patients enter a comprehensive treatment pathway, receiving integrated interventions based on the biopsychosocial model, including standardized pharmacotherapy, psychotherapy, physical therapy, and rehabilitation training. All treatments are delivered by a multidisciplinary team comprising psychiatrists, nurses, psychotherapists, and rehabilitation therapists, and are dynamically adjusted through daily ward rounds and regular case conferences. Discharge decisions are also structured, based on criteria such as symptom remission, reduced risk, and readiness of the support plan, and include systematic pre-discharge education and referral arrangements to ensure continuity of care.

In summary, the highly structured, multidisciplinary collaborative clinical practice model at this center is deeply rooted in national industry standards, international evidence-based guidelines, and documented internal quality control systems. This provides a practical basis for comparing and discussing the service utilization patterns observed in this study with those in other institutions operating at a similar level of standardization.


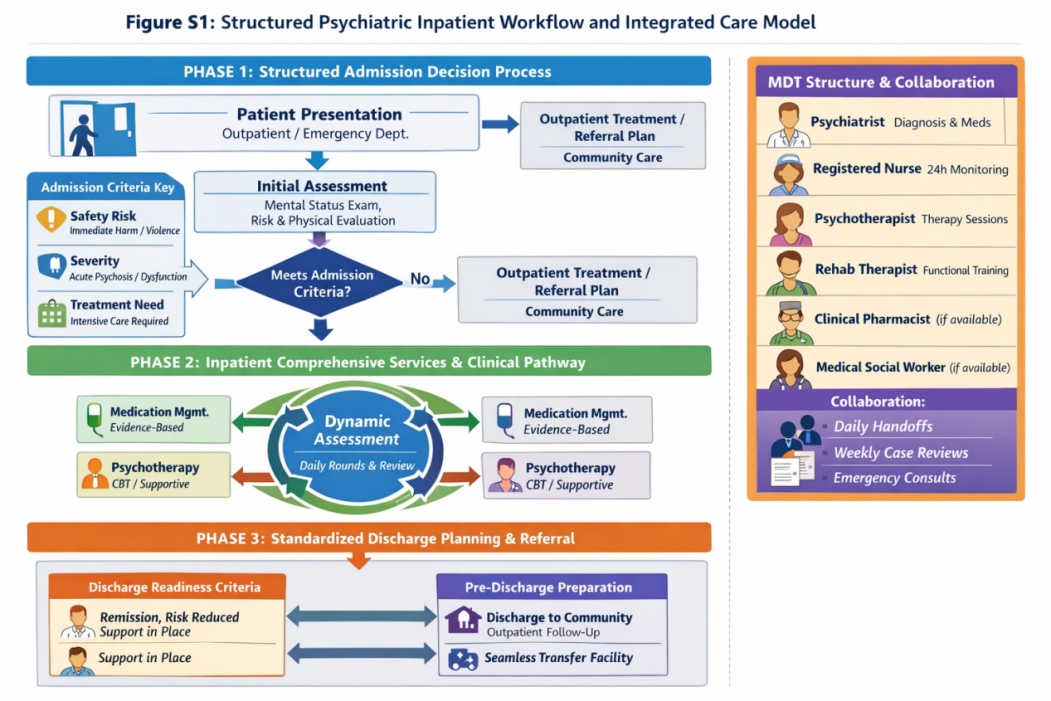


**Supplementary Figure S1.** Structured Psychiatric Clinical Workflow: Admission, Treatment, Discharge, and MDT Collaboration.

**3. Health Insurance Payment Structure, Reforms, and Potential Influences During the Data Collection Period**

The entire timeframe covered by this study (2017–2020) coincided with a critical phase of payment method reform within China’s basic medical insurance system. During this period, the universal social medical insurance system had been consolidated into two main schemes: Urban Employee Basic Medical Insurance and Urban and Rural Resident Basic Medical Insurance. Due to different funding sources, these two schemes have structural differences in benefit levels and reimbursement rates, which have been widely shown to be closely related to patterns of inpatient service utilization.

More importantly, the State Council and the Liaoning Provincial Government successively issued guiding plans to deepen payment method reform (State Council Document No. 55, 2017; Liaoning Government Document No. 97, 2017), initiating a shift from traditional fee-for-service payment to prospective payment models like “per-diem payment.” For psychiatric hospitalization, this reform aimed to guide hospitals to proactively optimize clinical pathways and control costs through a financial incentive mechanism of “retaining savings and sharing reasonable overruns.” Therefore, throughout the study period, the hospital’s management decisions were made under the clear expectation of this payment reform. Its choices regarding patient length of stay and service intensity may have been systematically influenced by this emerging incentive structure.

In conclusion, the policy context influencing these study’s findings has a dual character: first, the inherent differences in coverage levels between insurance types; and second, the comprehensive launch of payment method reform represented by “per-diem payment.” This context is crucial for assessing the external validity of the findings. Although specific payment standards vary by region, the paradigm shift “from fee-for-service to prospective payment” and “establishing value-based incentives” has become a shared national reform direction. Therefore, the underlying mechanisms of the service utilization patterns related to insurance type and payment reform expectations, revealed within the context of this study, hold significant reference value for understanding other Chinese regions undergoing similar reform processes.

**Supplementary Table 1.** ICD-10 Classification of Mental and Behavioral Disorders.

| **Category** | **Description** | **ICD-10 codes** |
| --- | --- | --- |
| F0 Organic | organic, including symptomatic, mental disorders. | F00–F09 |
| F1 Substance Misuse | mental and behavioral disorders caused by psychoactive substance use. | F10–F19 |
| F2 Schizophrenia and Psychotic | schizophrenia and schizotypal and delusional disorders. | F20–F29 |
| F3 Mood | mood (affective) disorders. | F30–F39 |
| F4 Neurotic, Stress and Anxiety | neurotic, stress-related and somatoform disorders. | F40–49 |
| Other Diagnosis | any other disorder not contemplated by the previous categories. | F50–99 |

**Supplementary Table 2.** Variable Definitions and Coding.

| **CODE** | **Name** | **Type** | **Classification and value assignment** |
| --- | --- | --- | --- |
| *Y* | total_hospitalization_expense | Continuous | Logarithmically transformed values, CNY (￥) |
| *X_1_* | gender | Categorical | Male=0, Female=1 |
| *X_2_* | age | Continuous | Years (18–100) |
| *X*_3_ | ethnicity | Categorical | Han Chinese=(0, 0, 0) , Manchu=(1, 0, 0), Hui=(0, 1, 0), Other=(0, 0, 1) |
| *X*_4_ | payment_method | Categorical | Urban Employee Basic Medical Insurance=(0, 0, 0, 0),  Urban and Rural Residents Basic Medical Insurance=(1, 0, 0, 0),  Rural Cooperative Medical System=(0, 1, 0, 0)  Out-of-Pocket=(0, 0, 1, 0),  Other=(0, 0, 0, 1) |
| *X*_5_ | job | Categorical | Employed=(0, 0, 0) , Retired=(1, 0, 0) ,  Unemployed=(0, 1, 0) , Other =(0, 0, 1) |
| *X_6_* | marital_status | Categorical | Unmarried=(0, 0, 0, 0) , Married=(1, 0, 0, 0) ,  Divorced=(0, 1, 0, 0) , Widowed=(0, 0, 1, 0) , Other=(0, 0, 0, 1) |
| *X_7_* | aCCI | Categorical | 0=(0, 0), 1= (1, 0), >1=(0, 1) |
| *X_8_* | diagnosis_groups | Categorical | F0 Mood affective disorder =(0, 0, 0, 0, 0) ,  F1 Schizophrenia schizotypal and delusional =(1, 0, 0, 0, 0) ,  F2 Adult personality and behaviour =(0, 1, 0, 0, 0) ,  F3 Neurotic stress related and somatoform=(0, 0, 1, 0, 0) ,  F4 Psychoactive substance use=(0, 0, 0, 1, 0) ,  Other diagnosis=(0, 0, 0, 0, 1) |
| *X_9_* | mortality_risk_level | Categorical | Low=1, Mid=2, High=3 |
| *X_10_* | admit_type | Categorical | Emergency=(0,0), Outpatient=(1,0), Other=(0,1) |
| *X_11_* | total_admissions | Categorical | 1=1, 2-5=2, >5=3 |
| *X_12_* | length_of_stay | Continuous | Days |
| *X_13_* | readmission_plan | Continuous | 1=Yes, 2=No |
| *X_14_* | discharge_status | Continuous | Cured=(0, 0, 0), Improved=(1, 0, 0), Not Improved=(0, 1, 0), Other=(0, 0, 1) |
| *X_15_* | cost_efficiency_ratio | Continuous | Continuous variable |
| Note: The “Other” category primarily includes individuals not classified as “Employed,” “Retired,” or “Unemployed” in the electronic medical record system, such as the self-employed, freelancers, students, homemakers, and temporary or casual workers. | | | |

**Supplementary Table 3.** Final Model Hyperparameters.

| **Model** | **Search Space** | **Final Hyperparameters** |
| --- | --- | --- |
| OLS | N/A | N/A |
| Random Forest | mtry: {3,5,7,10};  min.node.size: {5,10,20};  n.trees: 500 | mtry=7, min.node.size=20, n.trees=500 |
| GBM | n.trees: {100,300,500};  depth: {3,5,7};  shrinkage: {0.01,0.1} | n.trees=500, depth=7, shrinkage=0.01 |
| XGBoost | nrounds: {100,300,500};  max_depth: {3,6,9};  eta: {0.01,0.1,0.3} | rounds=100, max_depth=3, eta=0.1 |
| MLP | size: {5,10,20};  decay: {0.001,0.01,0.1} | size=5, decay=0.1 |
| Note: All models were tuned using 5-fold cross-validation. Optimal parameters were selected based on minimum RMSE. | | |

**Supplementary Table 4.** Descriptive Statistics of Hospitalization Costs by Cluster.

| **cluster** | **N (%)** | **Mean±SD** | **Median (IQR)** | **Range (Min-Max)** |
| --- | --- | --- | --- | --- |
| Low-frequency short stay | 2197(64.69) | 25639.09**±**26182.2 | 16688.05  (7957.26-33464.78) | 24.2-152,095.0 |
| High-frequency short stay | 340(10.01) | 107655.16**±**34741.1 | 100877.29  (76614.26-135284.79) | 46,483.7-190,098.0 |
| Long-term intermittent | 182(5.36) | 185675.65**±**25662.74 | 183672.96  (168339.1-204434.59) | 115,689.9-251,235.0 |
| Long-term continuous | 677(19.94) | 312224.9**±**48147.87 | 323548.65  (298028.92-337450.82) | 4,256.6-446,632.0 |
| Note: SD = Standard Deviation; IQR = Interquartile Range. All costs are ￥. | | | | |

**Supplementary Table 5.** Baseline Characteristics by Trajectory Clusters.

| **Variable** | | **Overall**  **（n=3,396）** | **Low-frequency short stay**  **（n=2,197）** | **High-frequency short stay**  **(n=340)** | **Long-term intermittent**  **(n=182)** | **Long-term continuous (n=677)** | **p** |
| --- | --- | --- | --- | --- | --- | --- | --- |
| Gender | Male | 1554 (45.8) | 985 (44.8) | 141 (41.5) | 80 (44.0) | 348 (51.4) | 0.007 |
|  | Female | 1842 (54.2) | 1212 (55.2) | 199 (58.5) | 102 (56.0) | 329 (48.6) |  |
| Ethnicity | Han Chinese | 3182 (93.7) | 2016 (91.8) | 328 (96.5) | 178 (97.8) | 660 (97.5) | <0.001 |
|  | Manchu | 113 ( 3.3) | 101 ( 4.6) | 5 ( 1.5) | 2 ( 1.1) | 5 ( 0.7) |  |
|  | Hui | 34 ( 1.0) | 24 ( 1.1) | 2 ( 0.6) | 1 ( 0.5) | 7 ( 1.0) |  |
|  | Other | 67 ( 2.0) | 56 ( 2.5) | 5 ( 1.5) | 1 ( 0.5) | 5 ( 0.7) |  |
| Payment method | UEBMI | 1831 (53.9) | 884 (40.2) | 248 (72.9) | 146 (80.2) | 553 (81.7) | <0.001 |
|  | URRBMI | 763 (22.5) | 548 (24.9) | 75 (22.1) | 32 (17.6) | 108 (16.0) |  |
|  | RCMS | 240 ( 7.1) | 238 (10.8) | 1 ( 0.3) | 0 ( 0.0) | 1 ( 0.1) |  |
|  | Out-of-Pocket | 528 (15.5) | 506 (23.0) | 9 ( 2.6) | 1 ( 0.5) | 12 ( 1.8) |  |
|  | Other | 34 ( 1.0) | 21 ( 1.0) | 7 ( 2.1) | 3 ( 1.6) | 3 ( 0.4) |  |
| Job | Employed | 511 (15.0) | 353 (16.1) | 54 (15.9) | 19 (10.4) | 85 (12.6) | <0.001 |
|  | Retired | 561 (16.5) | 248 (11.3) | 58 (17.1) | 42 (23.1) | 213 (31.5) |  |
|  | Unemployed | 1426 (42.0) | 1015 (46.2) | 134 (39.4) | 64 (35.2) | 213 (31.5) |  |
|  | Other | 898 (26.4) | 581 (26.4) | 94 (27.6) | 57 (31.3) | 166 (24.5) |  |
| Marital status | Unmarried | 1537 (45.3) | 1017 (46.3) | 162 (47.6) | 65 (35.7) | 293 (43.3) | <0.001 |
|  | Married | 1121 (33.0) | 815 (37.1) | 106 (31.2) | 53 (29.1) | 147 (21.7) |  |
|  | Divorced | 479 (14.1) | 254 (11.6) | 46 (13.5) | 41 (22.5) | 138 (20.4) |  |
|  | Widowed | 146 ( 4.3) | 57 ( 2.6) | 12 ( 3.5) | 13 ( 7.1) | 64 ( 9.5) |  |
|  | Other | 113 ( 3.3) | 54 ( 2.5) | 14 ( 4.1) | 10 ( 5.5) | 35 ( 5.2) |  |
| Diagnosis groups | F0 | 188 ( 5.5) | 125 ( 5.7) | 19 ( 5.6) | 0 ( 0.0) | 44 ( 6.5) | <0.001 |
|  | F1 | 163 ( 4.8) | 142 ( 6.5) | 9 ( 2.6) | 3 ( 1.6) | 9 ( 1.3) |  |
|  | F2 | 2097 (61.7) | 1201 (54.7) | 215 (63.2) | 132 (72.5) | 549 (81.1) |  |
|  | F3 | 649 (19.1) | 502 (22.8) | 67 (19.7) | 32 (17.6) | 48 ( 7.1) |  |
|  | F4 | 242 ( 7.1) | 193 ( 8.8) | 25 ( 7.4) | 13 ( 7.1) | 11 ( 1.6) |  |
|  | Other | 57 ( 1.7) | 34 ( 1.5) | 5 ( 1.5) | 2 ( 1.1) | 16 ( 2.4) |  |
| aCCI | 0 | 1220 (35.9) | 1008 (45.9) | 98 (28.8) | 50 (27.5) | 64 ( 9.5) | <0.001 |
|  | 1 | 985 (29.0) | 632 (28.8) | 118 (34.7) | 55 (30.2) | 180 (26.6) |  |
|  | 2 | 1191 (35.1) | 557 (25.4) | 124 (36.5) | 77 (42.3) | 433 (64.0) |  |
| Mortality risk level | Low | 2870 (84.5) | 1970 (89.7) | 293 (86.2) | 146 (80.2) | 461 (68.1) | <0.001 |
|  | Mid | 481 (14.2) | 214 ( 9.7) | 44 (12.9) | 35 (19.2) | 188 (27.8) |  |
|  | High | 45 ( 1.3) | 13 ( 0.6) | 3 ( 0.9) | 1 ( 0.5) | 28 ( 4.1) |  |
| Admit type | Emergency | 56 ( 1.6) | 51 ( 2.3) | 5 ( 1.5) | 0 ( 0.0) | 0 ( 0.0) | <0.001 |
|  | Outpatient | 3321 (97.8) | 2140 (97.4) | 332 (97.6) | 181 (99.5) | 668 (98.7) |  |
|  | Other | 19 ( 0.6) | 6 ( 0.3) | 3 ( 0.9) | 1 ( 0.5) | 9 ( 1.3) |  |
| Total admissions | 1 | 1657 (48.8) | 1610 (73.3) | 42 (12.4) | 0 ( 0.0) | 5 ( 0.7) | <0.001 |
|  | 2-5 | 1225 (36.1) | 582 (26.5) | 282 (82.9) | 156 (85.7) | 205 (30.3) |  |
|  | >5 | 514 (15.1) | 5 ( 0.2) | 16 ( 4.7) | 26 (14.3) | 467 (69.0) |  |
| Age | | 47.00 [34.00, 57.00] | 43.00 [31.00, 55.00] | 48.50 [36.00, 58.00] | 50.00 [36.25, 58.75] | 54.00 [46.00, 62.00] | <0.001 |
| Total hospitalization expenses | | 35,826.30  [12,084.06, 159,097.01] | 16,688.05  [7,957.26, 33,464.78] | 100,877.29  [76,614.26, 135,284.79] | 183,672.96 [168,339.10, 2e+05] | 323,548.65  [3e+05, 337,450.82] | <0.001 |
| LOS | | 127.00  [41.00, 565.25] | 59.00  [27.00, 117.00] | 369.50  [262.00, 473.00] | 614.00  [585.00, 689.50] | 1,096.00  [1,075.00, 1,096.00] | <0.001 |
| Note: UEBMI: Urban Employee Basic Medical Insurance; URRBMI: Urban and Rural Residents Basic Medical Insurance; RCMS: Rural Cooperative Medical System; aCCI: aged-Charlson Comorbidity Index; LOS: Length of Stay | | | | | | | |

**Supplementary Table 6.** Performance of GBM models: Set A vs Set B.

| **Feature Set** | **N(Variables/Features)** | **R^2^** | **RMSE** | **MAE** |
| --- | --- | --- | --- | --- |
| Set A (Baseline only) | 10/27 | 0.35 (0.303-0.396) | 1.242 | 1.005 |
| Set B (Baseline + Cluster) | 11/31 | 0.712 (0.68-0.741) | 0.826 | 0.571 |
| ΔR² (trajectory contribution)=R^2^ (Set B)−R^2^ (Set A)=0.712−0.35=0.362 | | | | |

**Supplementary Table 7.** Sensitivity Analysis of Model Performance.

| **Analysis Plan** | **N (Total/Test)** | **R^2^** |
| --- | --- | --- |
| Main Analysis (70:30) | 3396/1016 | 0.35(0.303-0.396) |
| S1: F2 Subgroup Only | 2097/629 | 0.304(0.239-0.365) |
| S2: Excluding >95th percentile | 3226/966 | 0.312(0.264-0.358) |
| S3a: Split 80:20 (stratified) | 3396/676 | 0.392(0.336-0.441) |
| S3b: Split 60:40 (stratified) | 3396/1355 | 0.349(0.305-0.394) |
| S4a: Weighted Training (Unweighted Eval) | 3396/1016 | 0.315(0.268-0.358) |
| S4b: Weighted Training (Weighted Eval) | 3396/1016 | 0.307(0.197-0.409) |
| Note: All metrics were evaluated on the independent test set using the final GBM model (trained with Set A features). The R² was calculated on a logarithmic scale, and the 95% confidence interval (CI) was derived from 1,000 bootstrap resamples. | | |


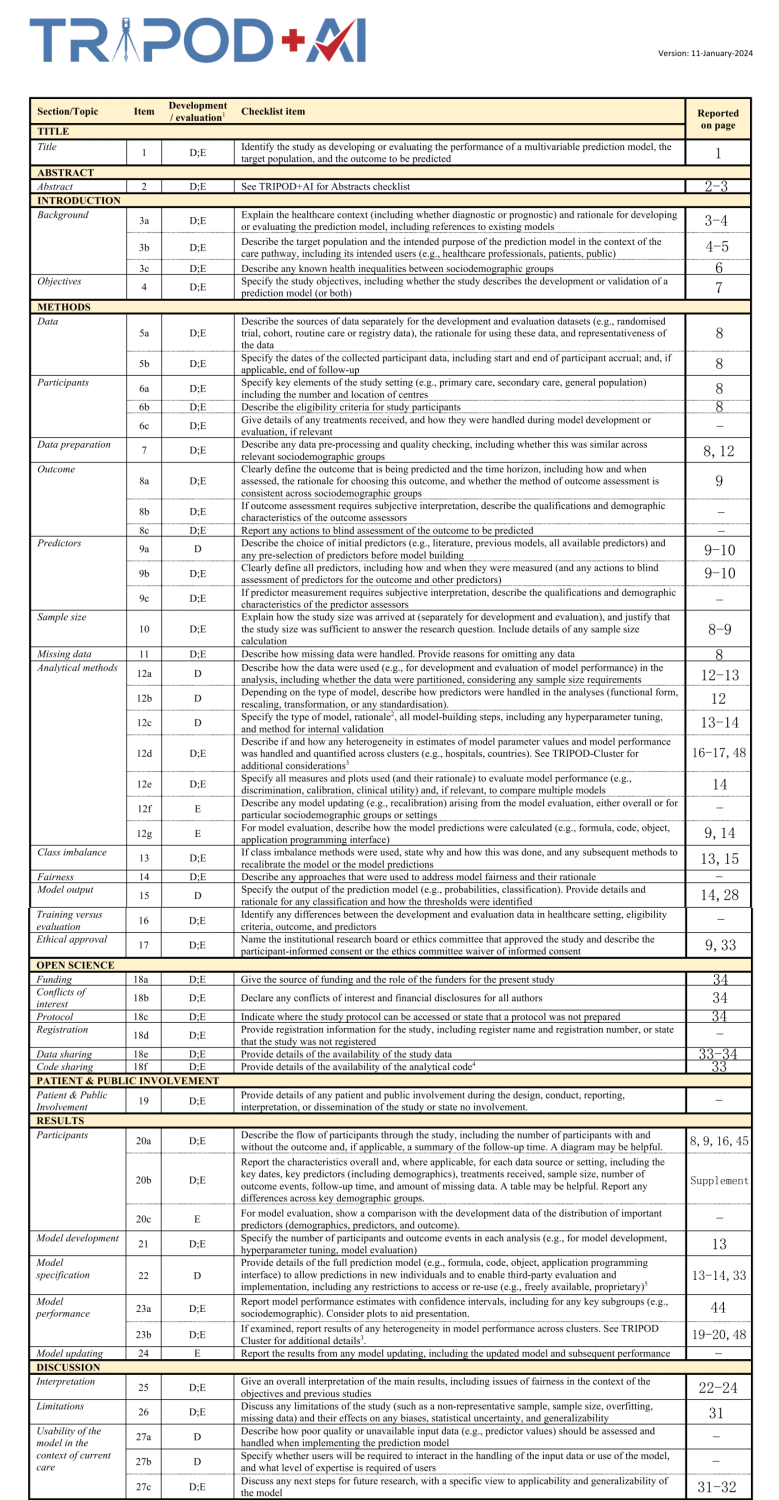


**Supplementary Figure 1.** TRIPOD + AI checklist.


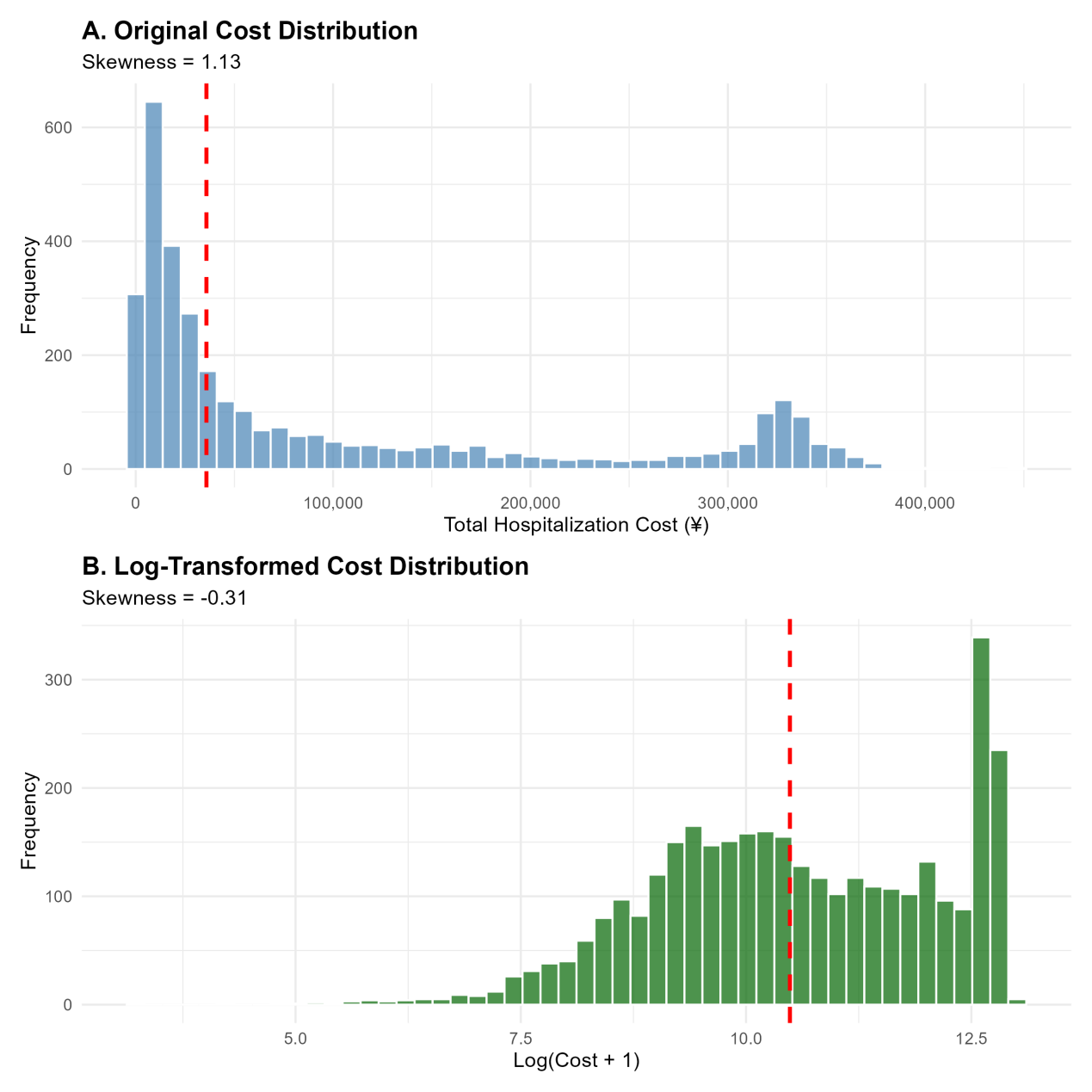


**Supplementary Figure 2.** Distribution of 3-Year Cumulative Hospitalization Costs.

**
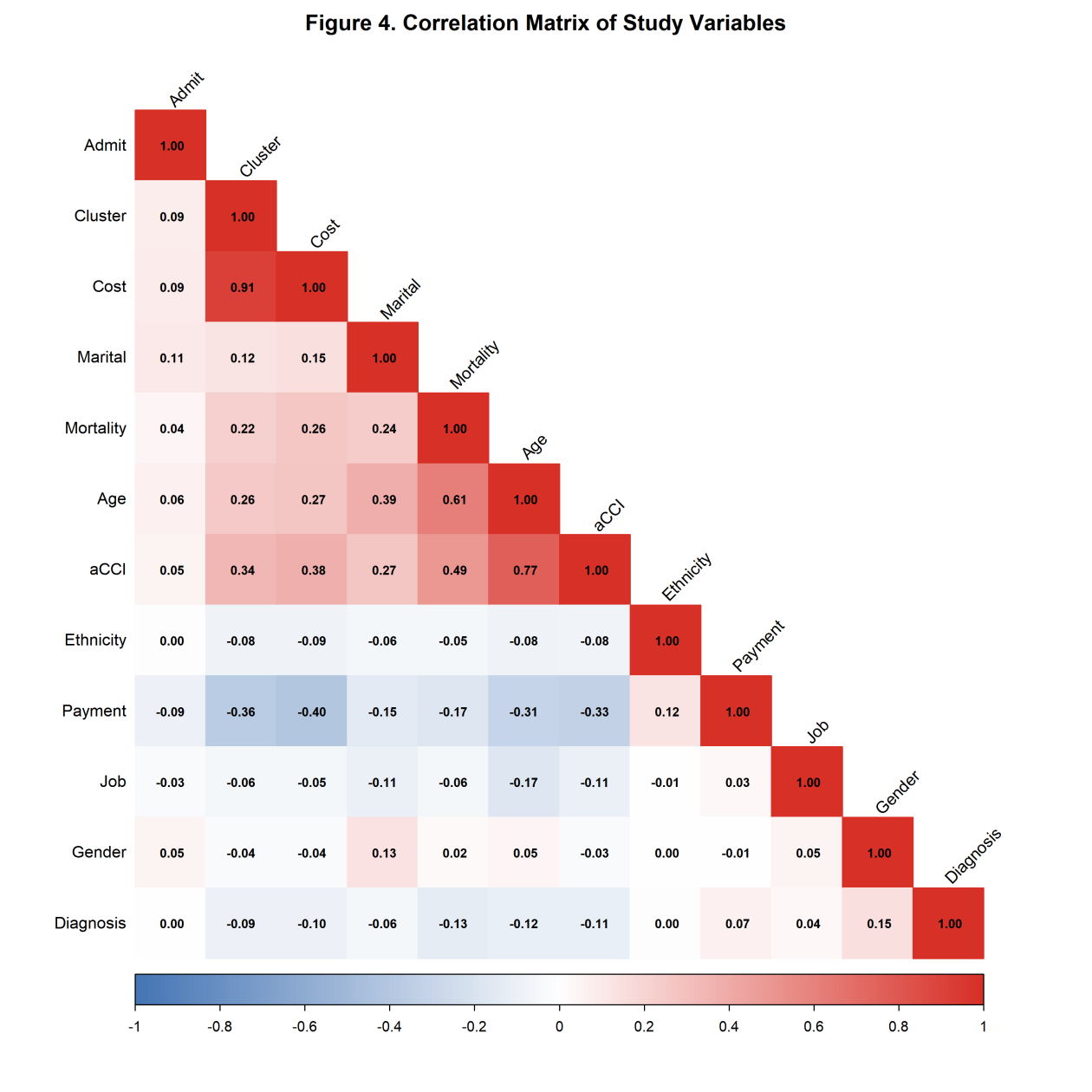
**

**Supplementary Figure 3.** Correlation matrix for all features in the dataset.

Note: Dark red indicates a strong positive correlation, white represents weak or no correlation, and dark blue denotes a strong negative correlation.


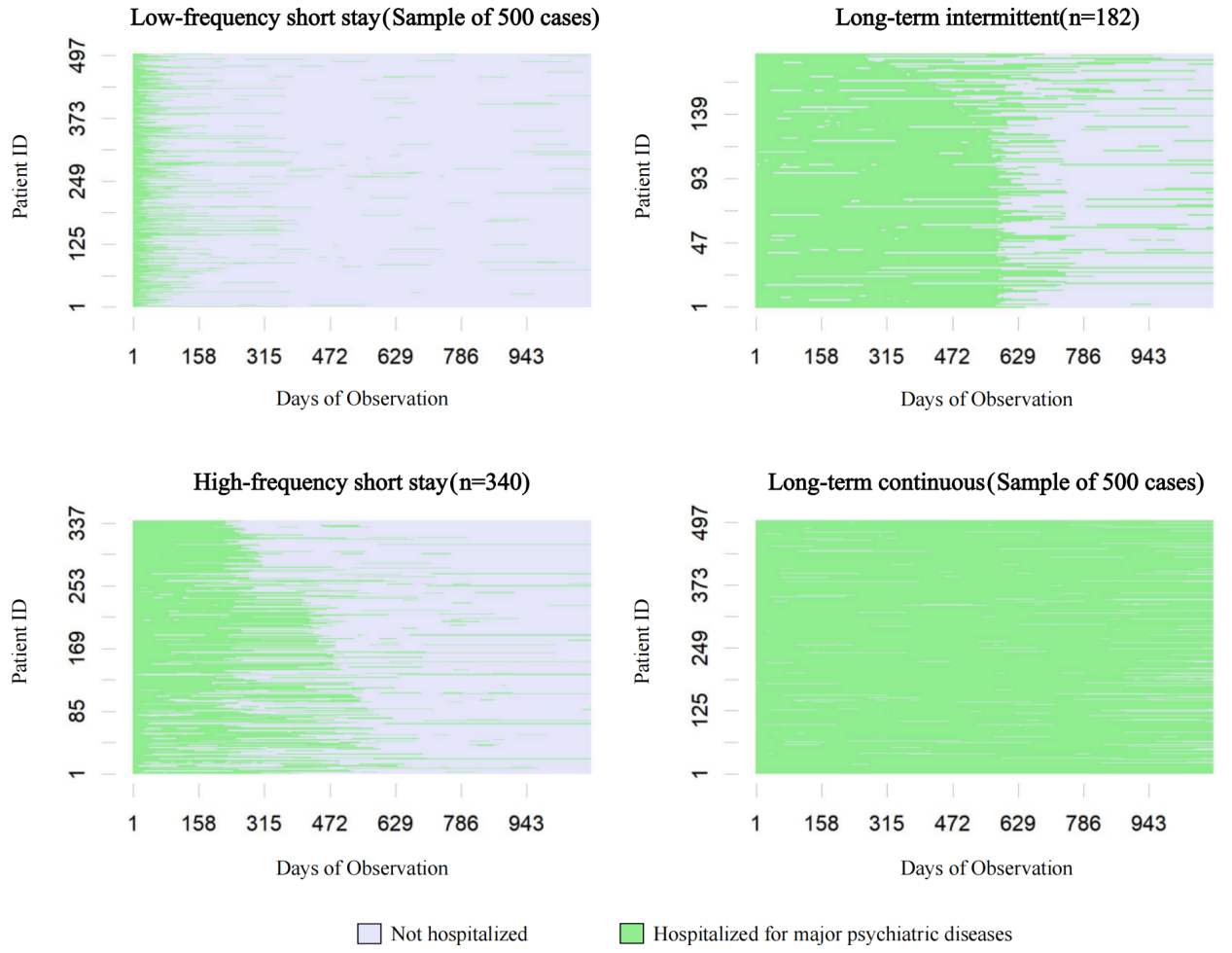


**Supplementary Figure 4.** Sequence visualization of the four hospitalization pattern clusters.

Note: This figure shows the hospitalization status of patients in each cluster during the 1,096 days following their first admission (states include: not hospitalized; hospitalized due to mental disorders).


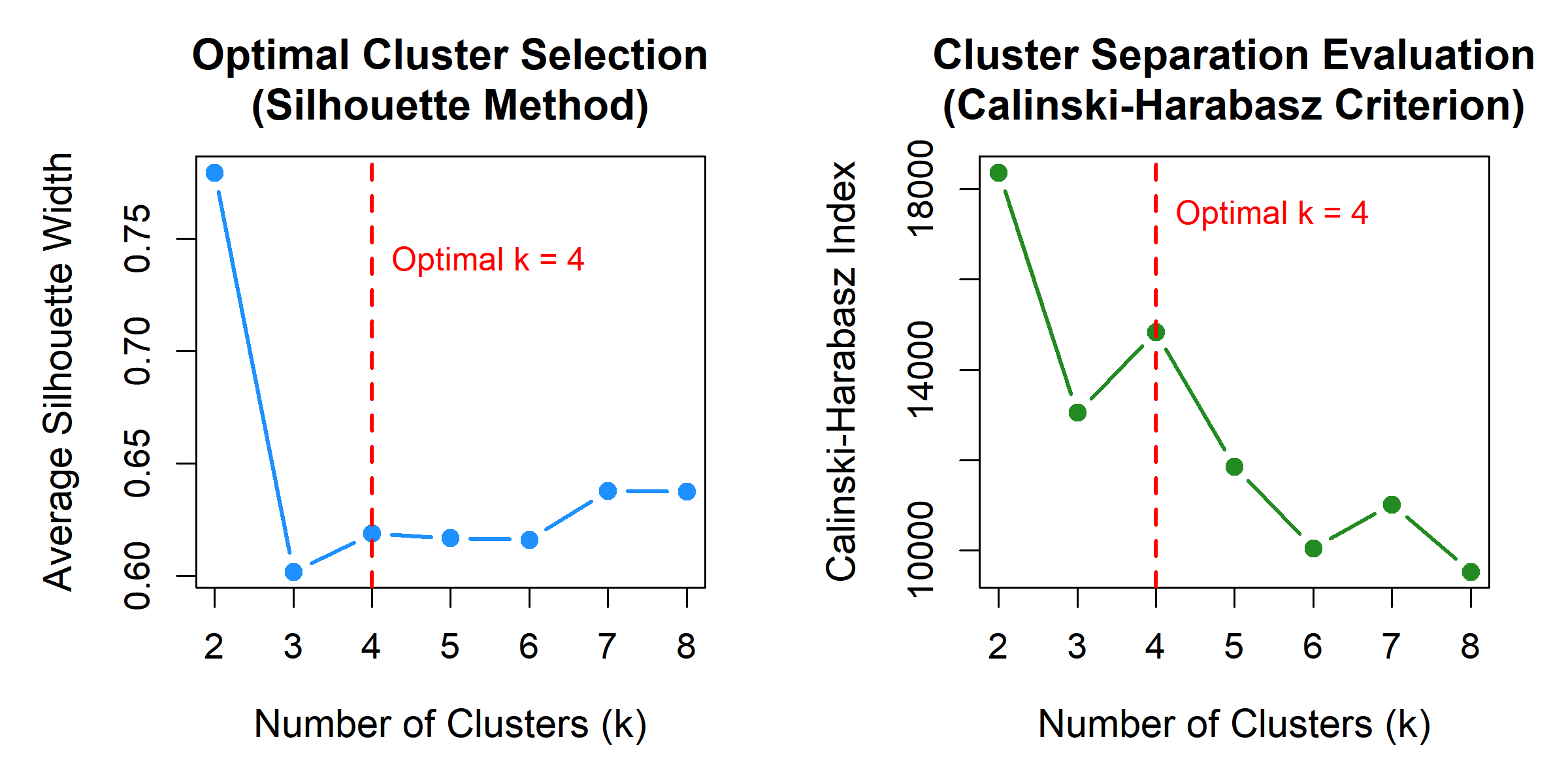


**Supplementary Figure 5.** Optimal Cluster Selection Based on Silhouette Width and Calinski-Harabasz Index.


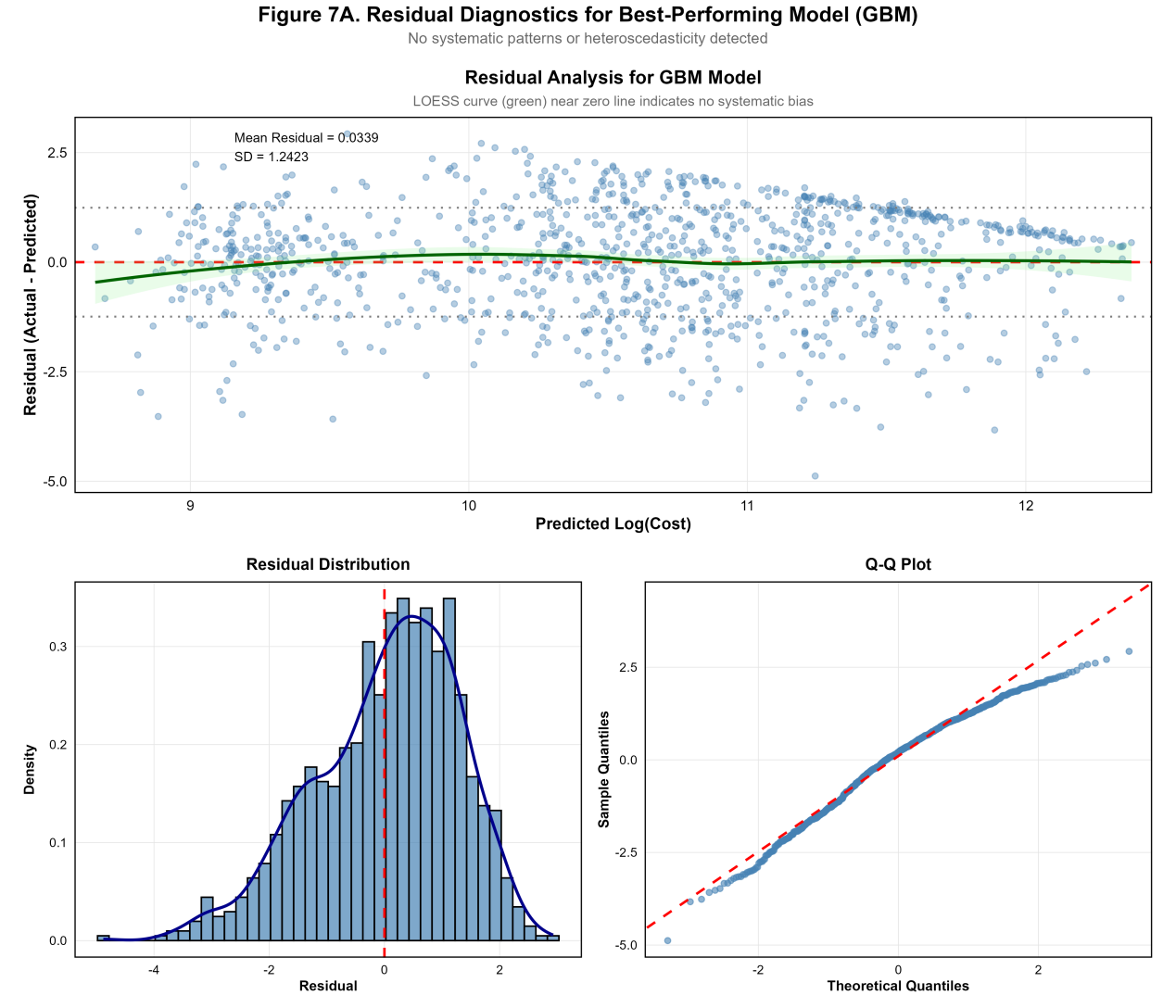


**Supplementary Figure 6.** Residual Diagnostics for Best-Performing Model (GBM).

Note: Comprehensive residual diagnostics for the GBM model. (A) The residual plot shows random scatter around zero with a LOESS curve near the horizontal axis. (B) The histogram and density plot indicate an approximately normal distribution. (C) Q-Q plot shows good agreement with theoretical normal distribution except in extreme tails.


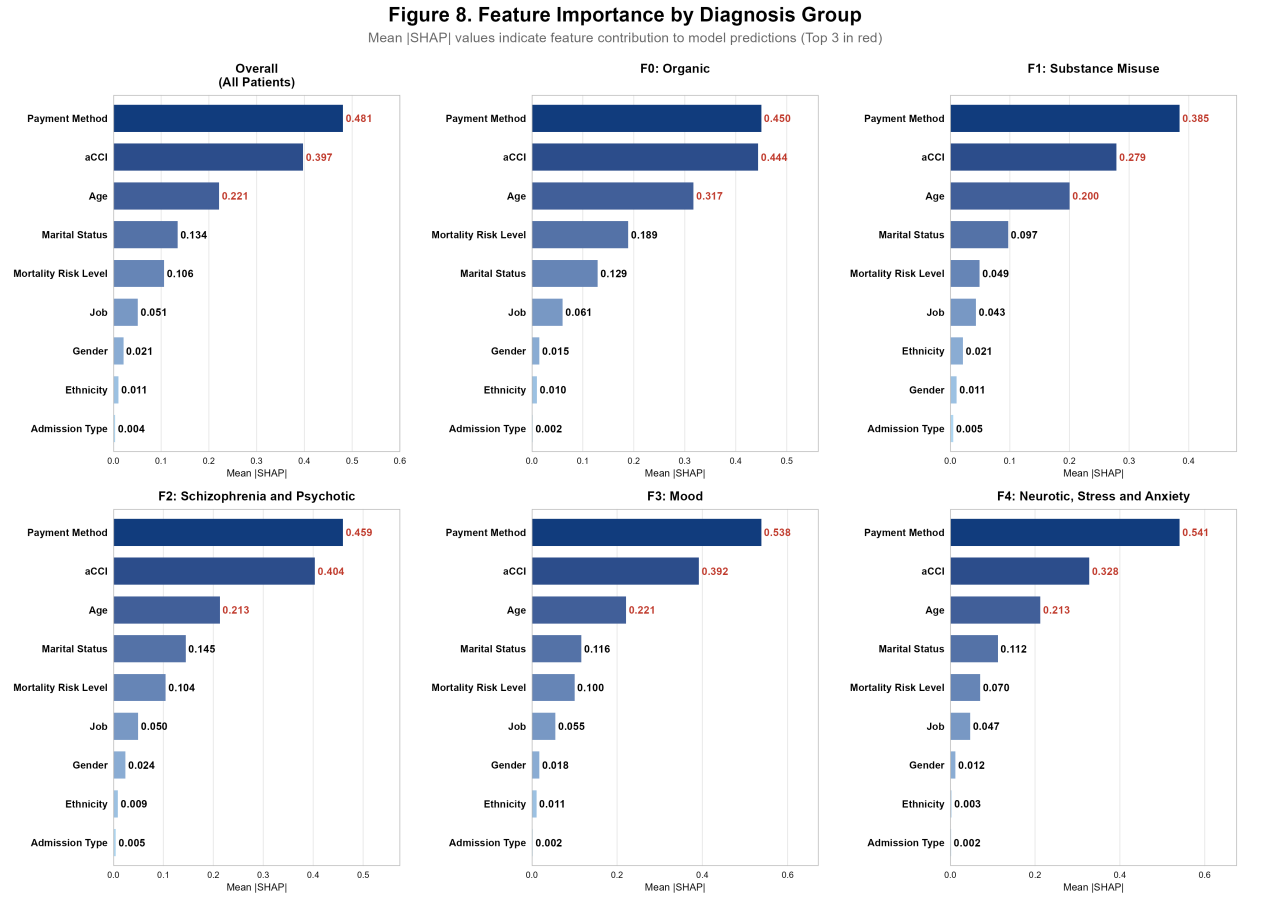


**Supplementary Figure 7.** Feature importance by Diagnosis Groups.

Note: The figure displays the mean SHAP values for each feature stratified by diagnostic subgroups and the overall cohort. Two metrics are shown per group: Mean |SHAP| (left column) representing the average absolute SHAP value, indicating feature importance magnitude; and Mean SHAP (right column) representing the average SHAP value, indicating the direction and strength of the feature’s contribution to the model output. Higher Mean |SHAP| values reflect greater impact on model predictions.
